# Adipokines at the Metabolic–Brain Interface: Therapeutic Modulation by Antidiabetic Agents and Natural Compounds in Alzheimer’s Disease

**DOI:** 10.3390/ph18101527

**Published:** 2025-10-11

**Authors:** Paulina Ormazabal, Marianela Bastías-Pérez, Nibaldo C. Inestrosa, Pedro Cisternas

**Affiliations:** 1 Facultad de Ciencias para el Cuidado de la Salud, Universidad San Sebastián, Lota 2465, Providencia, Santiago 7510157, Chile; paulina.ormazabal@uss.cl; 2 Centro de Investigación en Ciencias Biológicas y Químicas (CICBQ), Facultad de Medicina Veterinaria y Agronomía, Universidad de las Américas, Santiago 7500658, Chile; mbastias@udla.cl; 3 Núcleo de Investigación en Nutrición y Ciencias Alimentarias (NINCAL), Facultad de Salud y Ciencias Sociales, Universidad de las Américas, Santiago 7500658, Chile; 4 Centro de Excelencia en Biomedicina de Magallanes (CEBIMA), Universidad de Magallanes, Punta Arenas 6200000, Chile; ninestrosa@bio.puc.cl; 5 Facultad de Ciencias Biológicas, Pontificia Universidad Católica de Chile, Av. Bernardo O’Higgins 340, Santiago 8331150, Chile

**Keywords:** insulin resistance, adipose tissue dysfunction, metabolic inflammation, pharmacological interventions, GLP-1 receptor agonists, metformin, thiazolidinediones, SGLT2 inhibitors, DPP-4 inhibitors, phytochemicals, adiponectin, leptin, resistin, Alzheimer’s disease

## Abstract

The parallel global increase in obesity and Alzheimer’s disease (AD) underscores an urgent public health challenge, with converging evidence indicating that metabolic dysfunction strongly contributes to neurodegeneration. Obesity is now recognized not only as a systemic metabolic condition but also as a modifiable risk factor for AD, acting through mechanisms such as chronic low-grade inflammation, insulin resistance, and adipose tissue dysfunction. Among the molecular mediators at this interface, adipokines have emerged as pivotal regulators linking metabolic imbalance to cognitive decline. Adipokines are hormone-like proteins secreted by adipose tissue, including adiponectin, leptin, and resistin, that regulate metabolism, inflammation and can influence brain function. Resistin, frequently elevated in obesity, promotes neuroinflammation, disrupts insulin signaling, and accelerates β-amyloid (Aβ) deposition and tau pathology. Conversely, adiponectin enhances insulin sensitivity, suppresses oxidative stress, and supports mitochondrial and endothelial function, thereby exerting neuroprotective actions. The imbalance between resistin and adiponectin may shift the central nervous system toward a pro-inflammatory and metabolically compromised state that predisposes to neurodegeneration. Beyond their mechanistic relevance, adipokines hold translational promise as biomarkers for early risk stratification and therapeutic monitoring. Importantly, natural compounds, including polyphenols, alkaloids, and terpenoids, have shown the capacity to modulate adipokine signaling, restore metabolic homeostasis, and attenuate AD-related pathology in preclinical models. This positions adipokines not only as pathogenic mediators but also as therapeutic targets at the intersection of diabetes, obesity, and dementia. By integrating mechanistic, clinical, and pharmacological evidence, this review emphasizes adipokine signaling as a novel axis for intervention and highlights natural compound-based strategies as emerging therapeutic approaches in obesity-associated AD. Beyond nutraceuticals, antidiabetic agents also modulate adipokines and AD-relevant pathways. GLP-1 receptor agonists, metformin, and thiazolidinediones tend to increase adiponectin and reduce inflammatory tone, while SGLT2 and DPP-4 inhibitors exert systemic anti-inflammatory and hemodynamic benefits with emerging but still limited cognitive evidence. Together, these drug classes offer mechanistically grounded strategies to target the adipokine–inflammation–metabolism axis in obesity-associated AD.

## 1. Introduction

Obesity has emerged as one of the most pressing global health challenges of the 21st century [1]. Characterized by excessive accumulation of adipose tissue, it affects over 650 million adults worldwide and is a major risk factor for a range of cardiometabolic diseases, including type 2 diabetes, hypertension, and cardiovascular disorders [1,2,3]. Beyond its metabolic complications, obesity is now recognized as a significant contributor to global mortality rates. Recent research has also highlighted its detrimental impact on brain health, with growing evidence linking obesity to accelerated cognitive decline and an increased risk of developing neurodegenerative diseases, such as Alzheimer’s disease (AD) [4,5,6,7]. These findings underscore the urgent need to develop effective and early therapeutic strategies that address the multifaceted consequences of obesity across organ systems, including the brain.

In parallel, AD has become the most common neurodegenerative disorder and the leading cause of dementia worldwide [8,9]. It is marked by progressive memory impairment, cognitive dysfunction, and eventual loss of autonomy [10,11]. Current estimates suggest that more than 50 million individuals live with dementia, a figure expected to double by 2050 due to population aging [12]. Like obesity, AD is among the leading causes of death globally. The rising burden of AD further emphasizes the importance of identifying modifiable risk factors and implementing early interventions that may delay or prevent disease onset [13,14,15]. The convergence of these two life-threatening conditions, obesity and AD, reinforces the need for integrated, multidisciplinary approaches to understand their shared mechanisms with particular attention given to adipokines as predictive biomarkers and therapeutic targets, thereby guiding the development of novel preventive and treatment strategies.

Epidemiological data suggest that obesity, particularly during midlife, is associated with increased dementia risk in later years. This relationship is thought to be mediated by metabolic and vascular alterations, including chronic systemic inflammation, insulin resistance, and oxidative stress [6,7,16,17,18]. However, the underlying biological pathways that connect excess adiposity to neurodegeneration remain incompletely understood and may vary depending on the timing and chronicity of obesity exposure. Among the mechanistic links proposed are adipokines, hormone-like molecules secreted by adipose tissue that regulate metabolism, inflammation, and immune responses. As obesity progresses, the adipokine secretion profile becomes imbalanced, with increases in pro-inflammatory molecules such as resistin, tumor necrosis factor-alpha (TNF-α) and interleukin-6 (IL-6) among others and decreases in protective molecules like adiponectin, omentina-1, etc. [19]. Given their ability to cross the blood–brain barrier (BBB) and interact with neural cells, adipokines may represent key intermediaries through which peripheral metabolic dysfunction influences brain homeostasis [20,21]. These circulating adipokines access the brain via selective transport across the BBB, circumventricular regions, or indirectly through peripheral immune and vagal signaling, thereby shaping neuroimmune activation and metabolic tone [22].

This review explores the role of adipokines, mainly resistin and adiponectin, in mediating the effects of obesity-associated alterations, including low-grade chronic inflammation and impaired metabolism, on brain function. Emphasis is placed on their contributions to neuroinflammation and cerebral glucose metabolism, which are two processes central to AD pathogenesis and markedly dysregulated in the context of obesity. By synthesizing the current findings, we aim to clarify how these molecules may help explain the observed link between metabolic dysfunction and neurodegenerative disease. Special attention is given to emerging pharmacological and non-pharmacological strategies aimed at restoring adipokine balance, as well as to their potential utility as accessible peripheral biomarkers for early diagnosis and treatment monitoring. Finally, we discuss the current translational gap between preclinical and clinical research and underscore the need for future studies that incorporate sex, ethnicity, and metabolic status to guide personalized interventions. Together, these perspectives support the integration of adipokine biology into novel, metabolism-informed frameworks for AD prevention and care. The convergence of these two life-threatening conditions highlights adipokines as pivotal mediators and actionable biomarkers at the metabolic–brain interface.

## 2. Literature Search Methods

We performed a structured narrative search in PubMed/MEDLINE (January 2000–July 2025) using combinations of the following terms: adipokine, adiponectin, resistin, leptin, obesity, insulin resistance, Alzheimer’s disease, neuroinflammation, blood–brain barrier, GLP-1 receptor agonists, SGLT2 inhibitors, metformin, pioglitazone, natural compounds, diabetes and polyphenols. We included original preclinical and clinical studies, as well as recent systematic reviews and meta-analyses in English. Exclusion criteria: editorials, non-peer-reviewed sources, and studies not addressing adipokines or AD-relevant outcomes. Reference lists of key articles were hand-searched. Given the heterogeneity of study designs and outcomes, this review is qualitative and highlights convergent mechanisms and translational gaps. Limitations include possible publication bias and variability in adipokine assays across studies.

## 3. General Concepts of Alzheimer’s Disease

AD is a progressive, irreversible neurodegenerative disorder and the leading cause of dementia in older adults. Clinically, it is characterized by gradual cognitive decline, memory impairment, language disturbances, and deficits in executive function, ultimately leading to loss of independence [9,10]. AD accounts for approximately 60–80% of all dementia cases worldwide. Its prevalence increases with age, affecting between 6 and 7% of adults aged 65 and over and nearly 30% of those over 85, according to global estimates. With population aging, more than 110 million people are projected to be living with AD by 2050, posing enormous challenges for healthcare systems and underscoring the urgent need for preventive strategies and disease-modifying therapies [12,23,24,25].

The pathophysiology of AD is multifactorial, involving a complex interplay of genetic, environmental, and lifestyle factors. Familial or early-onset AD, which represents less than 5% of all cases, is primarily associated with mutations in genes such as amyloid precursor protein (APP) and presenilin 1 and 2 (PSEN1 and PSEN2). These genes encode key components of the γ-secretase complex, which, together with β-secretase (BACE1), is responsible for the proteolytic processing of APP. The sequential cleavage by BACE1 and γ-secretase generates Aβ peptides of varying lengths, most notably Aβ40 and Aβ42. While Aβ40 is the more abundant and relatively soluble form, Aβ42 contains two additional hydrophobic residues, making it more prone to misfolding and aggregation. Mutations in APP, PSEN1, or PSEN2 often lead to an increased Aβ42/Aβ40 ratio, favoring the accumulation of Aβ42 oligomers and fibrils. This process contributes to the formation of amyloid plaques, a central feature of AD pathology, ultimately triggering synaptic dysfunction, neuroinflammation, and progressive neurodegeneration [9,10,26,27]. Sporadic, or late-onset AD, the more common form, has a more complex etiology. The strongest known genetic risk factor for sporadic AD is the presence of the apolipoprotein E epsilon-4 allele (APOE ε4), which is associated with impaired Aβ clearance and increased inflammation [28,29,30].

Neuropathologically, AD is defined by two hallmark protein aggregates: extracellular Aβ plaques and intracellular neurofibrillary tangles composed of hyperphosphorylated tau protein [10]. These aggregates disrupt neuronal signaling, promote synaptic loss, and lead to neuronal death. In addition to these proteinopathies, widespread neuroinflammation, synaptic degeneration, and cerebral glucose hypometabolism are now recognized as central features of the disease process [31]. The role of neuroinflammation in AD has garnered significant attention in recent years. Initially viewed as a secondary response to Aβ and tau pathology, inflammation is now considered a key driver of disease progression. Activated microglia and astrocytes, the primary immune cells of the central nervous system (CNS), produce a range of pro-inflammatory cytokines, chemokines, and reactive oxygen species (ROS). While acute inflammation may have protective effects, chronic neuroinflammation contributes to synaptic dysfunction and accelerates neuronal loss [10,11,18,32,33,34].

Closely linked to neuroinflammation is the disruption of cerebral energy metabolism, particularly glucose utilization. The brain is a metabolically demanding organ that consumes approximately 20% of the body’s glucose supply despite comprising only 2% of total body weight [35]. Glucose is the primary energy source for neurons, and its metabolism is essential for maintaining synaptic function, plasticity, and neuronal survival [31,36,37]. In AD, positron emission tomography (PET) imaging studies using fluorodeoxyglucose (FDG) have revealed reduced glucose uptake in key brain regions, including the hippocampus and posterior cingulate cortex, even in preclinical stages [38]. Also, in AD, insulin signaling becomes impaired, a condition referred to as brain insulin resistance. Brain insulin resistance in AD means neurons and glia respond poorly to normal (or even elevated) insulin [39]. These dysfunctions in brain insulin have been related to reducing Aβ clearance and with an increase in tau hyperphosphorylation [40]. Importantly, this brain insulin resistance can occur without peripheral diabetes and correlates with cognitive deficits and AD biomarkers, highlighting a therapeutic target [41].

Emerging evidence suggests that brain insulin resistance and glucose hypometabolism may occur early in the disease course, even before the appearance of overt amyloid or tau pathology [42,43]. These metabolic abnormalities are increasingly being considered as potential initiating events rather than downstream consequences. The overlap between peripheral metabolic disorders, such as obesity and type 2 diabetes, and AD further supports the hypothesis that systemic metabolic dysfunction contributes to neurodegeneration through shared mechanisms.

**Figure 1:**
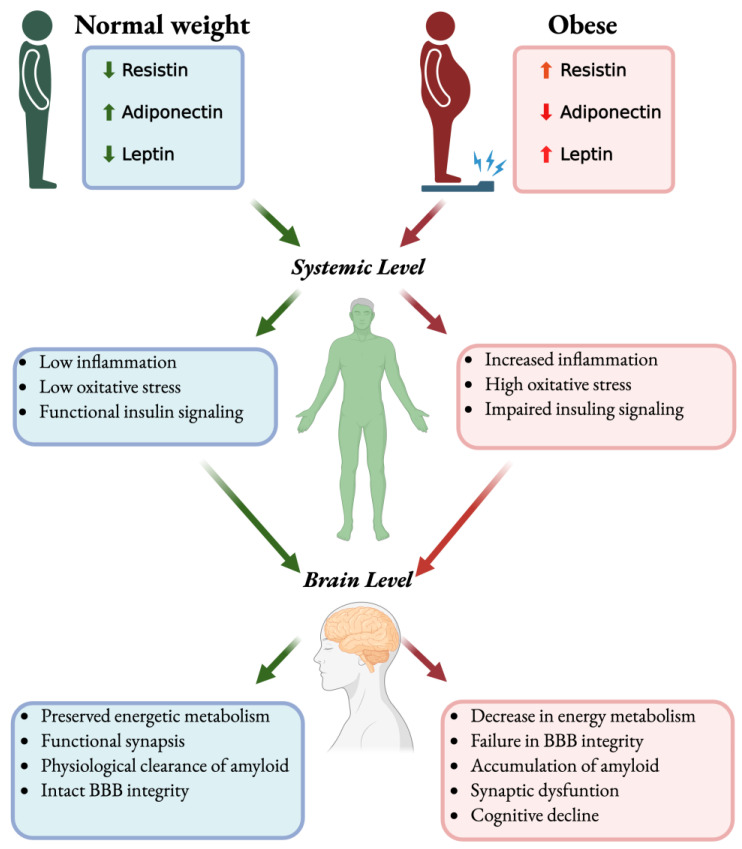
From adipokines to cognition: systemic–brain consequences of obesity. Schematic of the proposed pathway connecting adiposity, adipokines, and brain outcomes. In individuals with normal weight (**left**), lower resistin, higher adiponectin, and lower leptin are associated with low systemic inflammation and oxidative stress, together with preserved insulin signaling (green). These systemic features support brain homeostasis, adequate energy metabolism, functional synapses, physiological amyloid clearance, and an intact blood–brain barrier (BBB). In obesity (**right**), increased resistin and leptin with decreased adiponectin favor a pro-inflammatory, pro-oxidative milieu and impaired insulin signaling (red). This systemic state contributes to brain alterations consistent with AD pathophysiology, including reduced energy metabolism, BBB disruption, amyloid accumulation, synaptic dysfunction, and cognitive decline. Green arrows indicate protective influences; red arrows indicate deleterious influences (↑: increase; ↓: decrease).

Obesity is associated with chronic low-grade inflammation and altered secretion of adipokines, hormone-like molecules produced by adipose tissue, which can influence brain function. Elevated levels of pro-inflammatory cytokines such as TNF-α and IL-6, along with reduced anti-inflammatory adipokines like adiponectin, have been observed in both obese individuals and patients with AD (Figure 1). These circulating molecules can cross the BBB or act indirectly through endothelial activation and vagal nerve signaling, contributing to neuroimmune activation [22]. The concept of the ‘metabolic–inflammation axis’ in AD has led to increased interest in identifying biomarkers that reflect both metabolic and inflammatory states [44,45,46]. Understanding the interplay between inflammation, glucose metabolism, and adiposity offers a framework for integrating lifestyle-based interventions and metabolic therapies into AD prevention strategies. Approaches targeting insulin sensitivity, mitochondrial health, and inflammatory signaling may provide avenues to delay or prevent neurodegenerative changes. Furthermore, this perspective aligns with emerging paradigms of personalized medicine, emphasizing the need to tailor interventions based on an individual’s metabolic and inflammatory profiles [13,36,37,47,48,49] (Figure 1).

Key points, General Concepts of AD:

1. AD is the leading cause of dementia, with the burden rising sharply due to population aging.
2. Core pathology involves Aβ plaques and tau tangles, shaped by genetics and amplified by chronic neuroinflammation.
3. Early cerebral glucose hypometabolism and brain insulin resistance occur, often independent of peripheral diabetes, linking AD to systemic metabolic dysfunction.
4. Obesity-related cytokines and adipokine imbalance create a peripheral-to-central bridge that influences brain pathology.

## 4. Obesity as a Risk Factor for Alzheimer’s Disease

Obesity is a multifactorial, chronic disease characterized by excessive accumulation of adipose tissue and a disruption of metabolic homeostasis. Traditionally defined as a body mass index (BMI) equal to or greater than 30 kg/m^2^, obesity affects more than 650 million adults worldwide, and its prevalence continues to rise across all age groups [1].

In recent years, compelling evidence has emerged linking midlife obesity to an increased risk of late-life cognitive decline and AD. Several longitudinal population-based studies, including the Framingham Heart Study and the Honolulu-Asia Aging Study, have demonstrated that individuals with elevated BMI or central adiposity in midlife exhibit significantly greater risk of developing dementia decades later [50,51,52,53]. Central obesity, measured by waist-to-hip ratio, appears to be a stronger predictor of cognitive decline than BMI alone [4,7,16,54].

**Figure 2:**
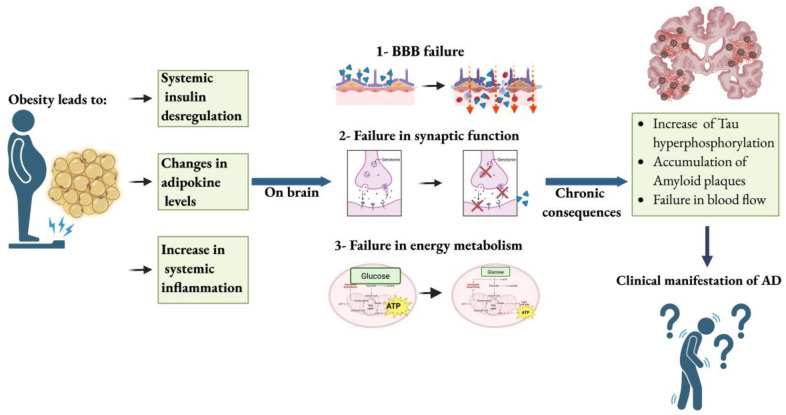
Obesity-to-brain cascade: systemic dysregulation to Alzheimer’s clinical expression. Proposed sequence connecting obesity with AD manifestations. Obesity promotes (**left**) systemic insulin dysregulation, changes in adipokine levels, and increased systemic inflammation. These systemic alterations impact the brain (center) through (**1**) blood–brain barrier (BBB) failure, (**2**) impaired synaptic function, and (**3**) deficits in energy metabolism. Over time (**right**), these changes converge on hallmark AD–related pathologies, including increased tau hyperphosphorylation, accumulation of amyloid plaques, and cerebral blood-flow disturbances, ultimately leading to the clinical manifestation of AD. Arrows indicate directionality across sequential levels (systemic → brain → chronic consequences).

While the association between obesity and dementia is robust in midlife, its interpretation in late life is more complex. Weight loss is often observed during the pre-symptomatic phase of AD, likely due to changes in appetite regulation, olfactory dysfunction, and systemic catabolism [17,55]. This reverse causality complicates the interpretation of observational studies and underscores the need for life course approaches in assessing adiposity-related risk. Importantly, neuropathological studies have revealed that obesity is associated with increased Aβ plaque burden and tau pathology [56] (Figure 2).

The mechanisms by which obesity contributes to neurodegeneration are diverse, involving chronic systemic inflammation, altered lipid metabolism, oxidative stress, cerebrovascular dysfunction, and impaired insulin signaling. Central to this interaction is the role of adipose tissue as an active endocrine organ. Adipocytes and resident immune cells within adipose depots secrete a wide array of adipokines and pro-inflammatory cytokines, including TNF-α, IL-6, and resistin [57]. These pro-inflammatory molecules do not remain confined to peripheral tissues. Circulating cytokines and adipokines can cross the BBB once in the CNS, and they contribute to the activation of microglia and astrocytes, initiating and sustaining a chronic neuroinflammatory response [32,33,58,59]. This inflammatory state accelerates neurodegenerative cascades, including synaptic loss, axonal damage, and neuronal death.

As mentioned above, insulin resistance has profound implications for AD pathogenesis, particularly through its modulation of Aβ clearance via the insulin-degrading enzyme (IDE) [60,61]. In AD and obesity, brain insulin resistance denotes reduced neuronal and glial responsiveness to insulin, characterized by impaired IRS-1/PI3K-AKT signaling, increased inhibitory serine phosphorylation of IRS-1, and downstream overactivation of GSK3β that favors tau phosphorylation [39]. Concomitant deficits in GLUT3/GLUT4 trafficking and IDE competition with circulating insulin can aggravate Aβ accumulation, synaptic failure, and cognitive decline [41]. Under normal metabolic conditions, IDE hydrolyzes both insulin and Aβ peptides, maintaining homeostasis in peripheral tissues and within the brain’s extracellular environment [41]. In insulin-resistant states, chronic hyperinsulinemia competes for IDE’s proteolytic activity. Although brain insulin concentrations are low, rendering direct competitive inhibition unlikely, upstream signaling disruptions critically impair IDE functionality. Specifically, resistance to insulin receptor activation blunts PI3K-Akt signaling, increases GSK-3β activity, and leads to tau hyperphosphorylation; concurrently, reduced Akt activity suppresses IDE’s expression and catalytic efficiency. This dual impairment mechanism, less clearance and increased production of pathological Aβ, amplifies plaque accumulation, triggering a feed-forward cascade of neuroinflammation and cognitive decline [17,62,63]. Genetic and *post-mortem* analyses reinforce the mechanistic link between insulin resistance and Aβ burden. IDE gene polymorphisms are associated with elevated AD risk, and reduced IDE expression/activity correlates with increased Aβ deposition [64,65,66]. In early and moderate AD, IDE levels rise, perhaps as a compensatory response, but deterioration of insulin signaling in later stages leads to sharp IDE decline, coinciding with plaque accumulation. A multicohort study involving T2D, AD, and control individuals found that serum IDE positively correlates with insulin resistance markers (BMI, HbA1c, HOMA-IR) in diabetic patients but inversely correlates with HbA1c and triglycerides in AD-only patients, indicating metabolic dysregulation exerts stage-dependent effects on IDE [67,68].

Mechanistic studies in transgenic and insulin-resistant rodent models further illustrate this pathway: insulin-resistant brains exhibit reduced neuronal IDE activity, increased Aβ plaque load, impaired astrocytic and microglial clearance, neuroinflammation, and tau pathology [69,70]. For instance, Sirt3-deficient mice, mimicking metabolic syndrome, show decreased IDE abundance, elevated amyloid accumulation, mitochondrial dysfunction, and heightened neuroinflammatory markers. Additionally, pharmacologic activation of IDE (e.g., via indole-derived compounds or tyrosine modification agents) restores Aβ degradation in vitro and slows cognitive decline in diabetic-AD mouse models [69,71,72].

Clinically, early indicators of insulin resistance, such as elevated triglyceride-glucose (TyG) index, predict accelerated cognitive decline and Aβ accumulation in presymptomatic stages in AD patients independent of genetic risk (e.g., APOE ε4), suggesting metabolic factors exacerbate amyloid pathology via IDE-linked pathways [73]. Collectively, these findings underscore a mechanistic model in which peripheral and central insulin resistance synergize to inhibit IDE-mediated Aβ clearance, promoting amyloidosis. Targeting IDE activity, either by enhancing its expression/stability or mitigating upstream insulin signaling defects, holds therapeutic promise. Ongoing efforts to develop IDE activators and insulin-sensitizing agents aim to alleviate amyloid burden and delay cognitive decline, positioning IDE-centered interventions as a critical component of future AD treatment in the obesity context.

Cerebral glucose hypometabolism is a defining feature of early AD. Positron Emission Tomography (PET) imaging studies using Fluorodeoxyglucose (FDG) consistently show reduced metabolic activity in AD-vulnerable regions, including the posterior cingulate cortex, parietotemporal association areas, and hippocampus [74,75,76]. Interestingly, similar hypometabolism patterns are observed in cognitively normal individuals with obesity, suggesting that metabolic dysfunction precedes and possibly contributes to pathological processes [77]. Structural neuroimaging studies have further revealed that individuals with obesity exhibit reductions in cortical thickness, particularly in frontal and temporal regions, as well as decreased hippocampal volume, key substrates of executive function and memory [78]. These anatomical changes are accompanied by cognitive deficits in processing speed, attention, and memory recall. In parallel, cerebrospinal fluid (CSF) studies and *postmortem* analyses have demonstrated that obesity is associated with elevated Aβ levels and phosphorylated tau [79,80,81].

Among the various mediators linking obesity to AD, adipokines have garnered particular attention due to their dual role in regulating inflammation and energy metabolism [19,20,21,82]. Adiponectin and resistin exhibit opposing effects on insulin signaling and neuroinflammation. Adiponectin, which is typically reduced in obesity, enhances insulin sensitivity, suppresses reactive oxygen species, and modulates anti-inflammatory pathways via AMP-activated protein kinase (AMPK) and peroxisome proliferator-activated receptor (PPAR) pathways [83,84]. In contrast, resistin levels are elevated in obesity and have been shown to promote insulin resistance and the release of pro-inflammatory cytokines such as TNF-α and IL-6. Resistin crosses the BBB and activates Toll-like receptor 4 (TLR4) signaling in microglia, amplifying neuroinflammatory cascades. Elevated plasma resistin has been associated with impaired memory performance and increased brain amyloid burden [85,86,87]. These findings support a model in which adipokine imbalance contributes to a shift toward a pro-inflammatory, metabolically dysfunctional CNS environment.

The systemic metabolic disturbances associated with obesity, such as hyperinsulinemia, dyslipidemia, and elevated free fatty acids (FFA), also have direct consequences for brain function. High circulating insulin levels may downregulate insulin transport across the BBB, exacerbating central insulin deficiency. Excessive FFA increases oxidative stress and impairs mitochondrial function in neurons, further amplifying energy deficits. Moreover, obesity-associated endothelial dysfunction can impair cerebral blood flow and compromise neurovascular coupling [88,89].

Given the multifaceted impact of obesity on AD-related pathology, interventions targeting adipose tissue function and adipokine profiles are being actively explored. Caloric restriction, exercise, and bariatric surgery have been shown to improve adipokine balance, reduce systemic inflammation, and enhance insulin sensitivity [90,91,92]. Metabolic inflammation also disrupts vascular–glymphatic homeostasis. Endothelial dysfunction and arterial stiffness diminish cerebral perfusion and impair neurovascular coupling, while glymphatic clearance of Aβ is reduced by insulin resistance and chronic low-grade inflammation, together accelerating proteinopathy and cognitive decline [93,94].

Preclinical models indicate that weight loss can reverse obesity-induced hippocampal inflammation and restore synaptic function [95]. Pharmacologic agents targeting adipokine signaling or enhancing insulin sensitivity are also under investigation. In addition to their mechanistic relevance, adipokines have potential clinical utility as biomarkers. Their presence in peripheral blood and CSF, combined with their sensitivity to metabolic changes, makes them attractive candidates for early risk stratification and therapeutic monitoring. Longitudinal studies examining the trajectories of adipokine levels in relation to cognitive decline are needed to validate their predictive value in clinical settings [96,97].

Key points, Obesity as a Risk Factor for AD:

1. Obesity in midlife strongly predicts later cognitive decline and higher amyloid/tau burden.
2. Adipose-driven inflammation and insulin resistance activate microglia, astrocytes and impair neurovascular–glymphatic homeostasis.
3. Imaging/biomarkers show obesity-linked cerebral hypometabolism, cortical/hippocampal atrophy, and adipokine imbalance, highlighting modifiable risk markers.
4. Lifestyle and metabolic interventions can ameliorate these pathways and are plausible disease-modifying strategies.

## 5. Neuroinflammation in Alzheimer’s Disease and Its Link to Obesity

Neuroinflammation is increasingly recognized not as a mere bystander, but as a central driver in AD. Chronic activation of microglia and astrocytes induces production of pro-inflammatory cytokines (e.g., TNF-α, IL-1β, IL-6), chemokines, complement proteins, and ROS. While transient immune activation may be neuroprotective, persistent inflammation impairs synaptic integrity, disrupts BBB function, and accelerates Aβ and tau pathologies [46,98,99].

Genetic studies underline the immune system’s involvement in AD. Genome-wide association studies have identified risk variants in microglia-regulating genes such as

Triggering receptor expressed on myeloid cells 2 (TREM2), Sialic acid-binding Ig-like lectin 3 (CD33), and Complement Receptor 1 (CR1), which modulate innate immune states and Aβ clearance [100,101]. Autopsy studies consistently document activated microglia clustering around amyloid plaques, expressing microglia markers like ionized calcium-binding adapter molecule 1 (IBA1) and Major Histocompatibility Complex class II (MHC-II) [102,103]. A newly recognized subset, disease-associated microglia (DAM), exhibits both protective and neurotoxic phenotypes, influenced by disease stage and local microenvironment [104]. Neuroinflammation and metabolic dysfunction are tightly interlinked. Cytokines such as TNF-α and IL-1β interfere with neuronal insulin signaling, reduce expression of GLUT1 and GLUT3, and impair mitochondrial bioenergetics through oxidative damage. This energy compromise leads to diminished ATP availability, vital for synaptic transmission and plasticity. Indeed, FDG–PET studies consistently detect early hypometabolism in vulnerable regions, including the hippocampus and posterior cingulate, even in asymptomatic individuals [31,46].

Neuroinflammation, often exacerbated by obesity-induced insulin resistance, plays a central and accelerating role in AD progression by influencing both amyloid and tau pathologies [105,106]. Obesity triggers chronic systemic inflammation, marked by increased pro-inflammatory cytokines, disruption of the BBB, and glial activation in the brain. Activated microglia and astrocytes, responding to obesity-associated inflammation, adopt prolonged pro-inflammatory states, releasing interleukin 1β (IL-1β), IL-6, TNF-α, reactive oxygen species (ROS), and proteases such as matrix metalloproteinases (MMPs) and cathepsins. This sustained glial activation directly impairs Aβ clearance: chronic neuroinflammation reduces microglial phagocytic efficiency, allowing Aβ plaques to accumulate. Simultaneously, pro-inflammatory cytokines exacerbate amyloidogenic processing by upregulating β and γ-secretase activity in neurons, further increasing Aβ production [107,108].

Inflammation also drives tau pathology. Cytokines such as IL-1β and TNFα activate tau kinases, including Glycogen synthase kinase-3 beta (GSK3β) and cyclin-dependent kinase 5 (CDK5), resulting in hyperphosphorylated tau, destabilization of microtubules, and neurofibrillary tangle formation. Experimental models of diet-induced obesity reveal elevated neuroinflammation accompanied by increased tau hyperphosphorylation and aggregation in transgenic mice [109,110]. There is compelling human evidence: mid-life visceral fat correlates with elevated PET markers of both Aβ and tau decades before cognitive symptoms, suggesting neuroinflammation links obesity to preclinical AD progression [111]. Furthermore, BBB breakdown induced by chronic systemic inflammation enables peripheral immune mediators to infiltrate the CNS, reinforcing neuroinflammatory loops that damage neurons and reduce metabolic resilience [112,113,114]. This inflammation–amyloid–tau nexus creates a vicious cycle: increased Aβ and tau exacerbate glial activation, which in turn promotes further pathological protein accumulation. Consequently, neuroinflammation represents more than a downstream effect; it is a driving force shaping both core pathologies of AD. Therapeutic strategies targeting upstream obesity-related inflammation, through weight loss, anti-inflammatory agents, or insulin-sensitizing drugs, may offer dual benefits by attenuating both amyloid and tau progression, thus slowing or preventing AD trajectory.

Obesity promotes a systemic-to-cerebral vascular cascade that culminates in endothelial dysfunction, impaired cerebral blood flow (CBF), and disrupted neurovascular coupling [115]. Insulin resistance blunts endothelial PI3K–Akt–eNOS signaling and nitric oxide (NO) bioavailability while upregulating endothelin-1–mediated vasoconstriction; concurrent dyslipidemia and hyperglycemia fuel oxidative stress and advanced glycation, further quenching NO [116]. At the microvascular level, pericyte loss/dysfunction and capillary rarefaction limit vasodilatory reserve, while astrocytic end-feet signaling (e.g., COX-2–dependent prostanoids, arachidonic metabolites) becomes maladaptive, decoupling neuronal activity from hemodynamic responses [117,118]. The net effect is chronic hypoperfusion and inefficient delivery of oxygen and glucose during cognitive demand, which synergize with amyloid- and tau-related toxicity to accelerate white-matter injury and cognitive decline. These vascular mechanisms provide a pathophysiologic bridge between obesity and neurodegeneration and suggest that interventions restoring endothelial NO signaling, dampening inflammation/oxidative stress, and stabilizing the BBB–pericyte–astrocyte axis could help re-establish neurovascular coupling in at-risk patients.

Obesity, marked by adipose hypertrophy and macrophage infiltration, induces a state of chronic, low-grade inflammation, often called “metaflammation” [119]. The inflamed adipose tissue releases cytokines and adipokines, which circulate systemically and cross or impact the BBB. These signals activate endothelial cells and perivascular macrophages, promoting microglial and astrocyte reactivity. Experimental models mirror these effects: rodents on high-fat diets exhibit early BBB disruption, increased expression of cytokines and complement factors, and hippocampal microglial activation, detectable as soon as two days after diet introduction [120,121]. High-fat diets also reduce hippocampal brain-derived neurotrophic factors (BDNFs), crucial in the survival, growth, and maintenance of neurons and impair synaptic proteins, leading to measurable cognitive deficits, highlighting the interconnection between neuroinflammation and metabolic impairment [122,123].

PET studies in Aβ-infused obese mice reveal simultaneous increases in the translocator protein (TSPO), a biomarker for glial activation and neuroinflammation, and FDG hypermetabolism, correlating with worse memory performance [124]. These findings suggest a dual metabolic and inflammatory response in early AD driven by obesity.

Oxidative stress, driven by ROS, damages mitochondrial and nuclear DNA, especially in neurons with high metabolic demands. Mitochondrial dysfunction leads to bioenergetic failure and triggers inflammasome activation. A proposed “lipid invasion” hypothesis further posits that dyslipidemia and BBB damage allow infiltration of free fatty acids (FFAs) and LDL into the brain, perpetuating inflammation and amyloid accumulation [125,126].

Age and obesity together exacerbate brain immune aging (“inflamm-aging”) and immunosenescence [125,127]. Middle-aged or aged rodents on high-fat regimens show exaggerated neuroinflammatory responses, particularly in the hippocampus, and greater cognitive impairment than younger counterparts [128,129]. This synergy likely reflects a reduced capacity for metabolic and immune resilience with age.

Human imaging confirms associations between obesity and brain dysfunction. Obese individuals without cognitive impairment exhibit hypometabolism in AD signature regions (hippocampus, posterior cingulate) on FDG–PET. A study comparing obese and diabetic patients found reduced FDG uptake in the temporal lobes even among metabolically healthy participants [130,131]. Moreover, visceral adiposity, specifically abdominal fat, predicts elevated amyloid and tau tracer binding in cognitively normal middle-aged adults [56,111]. Astrocyte-derived cholesterol supports neuronal Aβ generation via ApoE pathways. Cholesterol and FFAs can accumulate within the brain following BBB breakdown, triggering innate immune receptors and complement cascades [132,133,134]. This aligns with early inflammatory activation in AD and highlights a link between metabolic dysregulation and neurodegeneration.

Neuroinflammation remains a modifiable target, as shown by nonsteroidal anti-inflammatory drugs trials (though results depend heavily on timing) [135,136,137]. Exercise reduces microglial proliferation and inflammatory gene expression in aging models. Animal studies demonstrate that caloric restriction, weight loss, or pharmacological agents aimed at adjusting adipokine levels, blocking TLR4, or enhancing adiponectin receptor signaling can reduce glial reactivity, restore metabolic function, and improve cognition [138,139]. The convergence of systemic inflammation, brain metabolism, and adipokine dysregulation defines a nexus linking obesity to AD. Neuroinflammation, metabolic dysfunction, and altered adipokine signaling act synergistically to disrupt synaptic and neuronal integrity. Interventions targeting this axis, through diet, lifestyle modification, and selective pharmacology, hold promise, particularly if implemented early in obesity or metabolic syndrome. Further integration of translational biomarkers (PET, blood adipokines, genetic risk scores) will be vital for identifying optimal windows and tailoring preventative strategies.

Key points of Neuroinflammation and Obesity in AD:

1. Neuroinflammation is a disease driver: microglia/astrocyte activation accelerates Aβ/tau pathology and disrupts synapses and the BBB.
2. Genetic signals underscore causal involvement of immune pathways in AD.
3. Obesity amplifies neuroinflammation via systemic cytokines, insulin resistance, BBB leakage, and neurovascular–glymphatic dysfunction.
4. Imaging/biomarker evidence converges on this nexus and identifies modifiable targets.

## 6. Adipokines and Their Relevance in the Brain

Adipokines are bioactive peptides predominantly secreted by adipose tissue that serve as critical regulators of systemic and central processes, including metabolic homeostasis, inflammation, and neuronal function [20,82]. These molecules exert their actions through autocrine, paracrine, and endocrine mechanisms, with circulating levels tightly modulated by nutritional status, adiposity, and inflammatory signals. The capacity of several adipokines to cross the BBB or be synthesized locally within the brain has positioned them as central mediators of the crosstalk between peripheral metabolism and CNS function [20,82,140] (Table 1 and Figure 2).

Leptin, a hormone secreted primarily by white adipose tissue, exerts neuroprotective effects in AD through multiple mechanisms that are disrupted in obesity-induced leptin resistance. Under normal conditions, leptin crosses the BBB and binds to hippocampal and cortical leptin receptor (Ob-R) to activate pro-survival cascades, including JAK2-STAT3, PI3K-Akt, and MAPK, enhancing synaptic plasticity and long-term potentiation while reducing β-secretase activity and oxidative stress [153,154]. In addition to supporting neuronal survival, leptin directly promotes Aβ clearance and reduces tau phosphorylation in animal AD models [155,156,157,158]. For instance, chronic leptin treatment in transgenic APP/PS1 mice (mice with high levels of Aβ) decreased brain Aβ burden, lowered hyperphosphorylated tau, improved microglial responses, and rescued memory and synaptic function [159]. However, obesity induces leptin resistance, characterized by impaired BBB transport, saturated Ob-R signaling, and induction of negative regulators such as SOCS3, PTP1B, and TCPTP, often in an inflammatory milieu. Elevated systemic inflammation and elevated triglycerides compromise leptin’s neuroprotective entry and function, weakening its inhibition of amyloidogenic pathways and tau kinases like GSK-3β. Human cohort data from JAMA Network indicate that higher plasma leptin is inversely correlated with both Aβ and tau PET load in older adults, suggesting a protective role, yet this benefit is likely lost under leptin resistance [160,161].

Recent mechanistic studies have revealed that leptin modulates neuroinflammation by reducing microglial secretion of IL-1β, IL-6, and TNF-α, thereby indirectly stabilizing amyloid and tau pathology [159,162]. Recently, inflammatory paths study highlights leptin’s ability to buffer adipose-derived inflammation before it impairs cognition. Translational hopes from Dundee researchers illustrate that leptin-derived peptide fragments protect synaptic function by blocking Aβ and tau toxic effects, affirming hormone-based therapeutic potential, although full efficacy awaits overcoming resistance.

Adiponectin, in contrast, exhibits an inverse relationship with fat mass and is generally considered neuroprotective. It circulates in various multimeric forms, low, medium, and high molecular weight, with different biological activities, and exerts its effects via Adiponectin Receptor 1 and 2 (AdipoR1 and AdipoR2, respectively) receptors expressed in neurons, astrocytes, and microglia. Adiponectin activates AMPK and PPAR-α signaling pathways, leading to improved insulin sensitivity, mitochondrial biogenesis, and reduced oxidative stress [85,86]. In the CNS, adiponectin has been shown to enhance glucose uptake, support neuronal viability, and reduce inflammatory signaling. Preclinical studies have demonstrated that adiponectin administration in AD models reduces Aβ deposition, attenuates tau hyperphosphorylation, and improves memory performance. Interestingly, despite these benefits, clinical studies have yielded conflicting results regarding plasma adiponectin levels in AD patients, with some reports indicating elevated levels in individuals with dementia, possibly reflecting a compensatory response to neurodegeneration or systemic inflammation [144,163,164,165]. Nonetheless, the potential of adiponectin as a therapeutic molecule remains compelling, especially in its ability to modulate both peripheral insulin resistance and central inflammation.

Resistin, originally identified in rodents as a link between obesity and insulin resistance, is primarily secreted by macrophages in humans and functions as a pro-inflammatory cytokine [85,166]. It binds to receptors such as TLR4 and Adenylyl cyclase-associated protein 1 (CAP1), initiating factor nuclear κB (NF-κB)-dependent transcription of cytokines, including TNF-α and IL-6. Elevated resistin levels have been observed in obesity, type 2 diabetes, and cardiovascular disease, as well as in neurodegenerative disorders [89,167,168]. In the brain, resistin impairs insulin signaling, promotes oxidative stress, and induces the release of pro-inflammatory mediators from microglia and astrocytes. These actions not only disrupt neuronal glucose metabolism but also create a neuroinflammatory milieu conducive to the progression of AD pathology [169,170,171]. Human studies have linked higher resistin levels with cognitive impairment, increased brain atrophy, and greater Aβ burden, although its specific mechanistic role in AD remains an area of active investigation.

Beyond these well-characterized adipokines, other molecules such as visfatin, apelin, omentin, and chemerin are increasingly being studied for their potential roles in neurobiology. For example, visfatin has insulin-mimetic effects and may influence neuronal energy metabolism, while apelin has been shown to modulate neuroinflammation and protect against excitotoxicity [84] (Table 1). Although data remain preliminary, these emerging adipokines may represent additional targets for therapeutic exploration. Adipokines not only influence neuronal function directly but also modulate cerebrovascular integrity. Adiponectin enhances endothelial nitric oxide synthase (eNOS) activity and reduces vascular inflammation, thereby maintaining BBB function [172]. Leptin affects endothelial permeability and may facilitate the transport of Aβ across the BBB. In contrast, resistin disrupts tight junction proteins, increases BBB permeability, and promotes vascular inflammation, facilitating the entry of peripheral inflammatory mediators into the brain [173,174]. The resultant breach of the BBB is a key event in the amplification of neuroinflammation and progression of AD.

One of the most critical functions of adipokines in the brain is the regulation of glucose metabolism, a process profoundly impaired in AD. Leptin and adiponectin enhance GLUT1 and GLUT3, improve mitochondrial efficiency, and stimulate the AMPK–Peroxisome proliferator-activated receptor gamma coactivator 1-alpha (PGC1α) axis, which is crucial for maintaining synaptic energy supply [175,176,177]. Conversely, resistin suppresses insulin receptor signaling, reduces Akt phosphorylation, and inhibits glucose uptake, exacerbating neuronal energy failure. These opposing effects are reflected in imaging studies showing hypometabolism in AD-vulnerable brain regions in individuals with obesity or insulin resistance. The modulation of neuronal bioenergetics by adipokines underscores their relevance in the early stages of cognitive decline. Adipokines also interact with the molecular machinery involved in amyloid and tau pathology. Leptin has been shown to reduce β-secretase activity, thereby decreasing Aβ production, and to promote Aβ clearance by enhancing phagocytosis. Adiponectin indirectly facilitates Aβ degradation by promoting anti-inflammatory microglial phenotypes. Resistin, by contrast, enhances β-secretase expression and contributes to tau hyperphosphorylation through stress kinase activation [164,178]. These findings suggest that the balance between protective and deleterious adipokines may shape the molecular landscape of neurodegeneration.

Therapeutic strategies targeting adipokine signaling are actively being developed. AdipoRon, a synthetic agonist of AdipoR1 and AdipoR2, has shown neuroprotective effects in preclinical models of AD, including reduced Aβ accumulation and improved synaptic function. PPAR-γ agonists such as pioglitazone, which enhance adiponectin sensitivity, have also demonstrated cognitive benefits in small clinical trials, though results remain inconclusive [144,164,179,180]. Intranasal delivery of leptin or insulin has been explored as a means of bypassing peripheral resistance and directly modulating CNS pathways [181,182]. On the other hand, pharmacological blockade of TLR4 or CAP1, aimed at reducing resistin signaling, represents a promising anti-inflammatory strategy [183,184]. In addition to pharmacological approaches, lifestyle interventions offer non-invasive means of modulating adipokine profiles. Regular physical activity, caloric restriction, and weight loss have been shown to increase adiponectin levels, reduce leptin resistance, and lower systemic resistin. These changes are associated with improved cognitive function, reduced neuroinflammation, and preserved hippocampal volume in at-risk populations. Notably, the timing of intervention appears critical, as early metabolic modulation may have more profound effects on long-term brain health than late-stage correction.

While adiponectin and GLP-1–based strategies are biologically compelling, their translation to older adults with AD warrants caution. Although adiponectin exerts neuroprotective and insulin-sensitizing actions, clinical studies have reported heterogeneous circulating levels in dementia, including elevations in established AD, a phenomenon sometimes interpreted as a compensatory response or ‘adiponectin resistance [185,186]. This so-called adiponectin paradox has also been linked to higher all-cause and cardiovascular mortality in frail elders, underscoring the need for age-, stage-, and comorbidity-aware stratification when considering adiponectin-targeted therapies. GLP-1 receptor agonists, which show cognitive signals in metabolic populations, can cause gastrointestinal intolerance, volume depletion/dehydration, and unintended weight loss, adverse effects that may aggravate sarcopenia or orthostatic symptoms in adults ≥70 years [187,188]. Accordingly, trials in AD should incorporate careful dose-titration, hydration/renal monitoring, and nutritional surveillance, and should prespecify analyses by age, BMI, and frailty indices.

In terms of diagnostics, adipokines are attractive candidates for biomarker development due to their detectability in peripheral blood and CSF. Combinations of leptin, adiponectin, resistin, and other inflammatory markers may enhance early detection of individuals at risk for AD, especially when integrated with neuroimaging and cognitive assessments [189,190]. However, standardization of assays, understanding of isoform-specific effects, and adjustment for confounding variables such as age, sex, and comorbidities remain challenges for clinical translation (Table 2).

Key points, Adipokines and the Brain:

1. Adipokines bridge peripheral metabolism and CNS function.
2. Leptin is neuroprotective, but leptin resistance in obesity diminishes central signaling efficacy. Adiponectin enhances insulin sensitivity and is anti-inflammatory; dementia studies show heterogeneous levels. Resistin is pro-inflammatory, impairs insulin signaling, and compromises BBB integrity.
3. Adipokine imbalance disrupts cerebral glucose metabolism, amplifies neuroinflammation, and promotes Aβ/tau pathology.

## 7. Resistin and Adiponectin: Opposing Adipokines Forces in Alzheimer’s Disease

Obesity induces profound alterations in adipose-derived signals, among which resistin and adiponectin emerge as antagonistic regulators of inflammation, metabolism, and neurodegeneration. Their divergent roles significantly shape the brain’s vulnerability or resilience to AD.

Resistin, predominantly produced by macrophages and mononuclear immune cells in humans, increases substantially in obesity and metabolic syndrome [166]. Elevated resistin binds TLR4 and CAP1, triggering downstream pathways including NF-κB and ERK1/2. This cascade drives the production of pro-inflammatory cytokines, such as TNF-α and IL-6, and disrupts IRS-1 phosphorylation, impairing Akt activation [166]. The net effect is neuronal insulin resistance, reduced glucose uptake, and mitochondrial dysfunction. In vitro and in vivo studies demonstrate that resistin exposure increases oxidative stress markers, decreases ATP production, and impairs synaptic plasticity in hippocampal neurons [197]. Within the CNS, resistin further exacerbates pathology by directly activating glial cells. Microglia exposed to resistin upregulate inflammatory mediators (e.g., IL-1β, IL-18), enhance ROS production, and adopt [197,198,199,200] a phagocytic but neurotoxic phenotype. Astrocytes similarly increase secretion of cytokines and chemokines, contributing to a sustained neuroinflammatory milieu. Mechanistically, resistin also upregulates β-site amyloid precursor protein cleaving enzyme 1 (BACE1), accelerating Aβ generation, and it promotes tau phosphorylation via GSK-3β activation. In both rodent models and human *postmortem* brain tissue, resistin co-localizes with Aβ plaques and tau tangles; higher resistin levels in plasma and CSF correlate with hippocampal atrophy, cognitive impairment, and faster functional decline [201,202]. Beyond direct neural effects, resistin contributes to endothelial dysfunction and BBB disruption. By interfering with tight junction proteins (e.g., claudin-5, occludin) and increasing matrix metalloproteinase (MMP) activity, resistin facilitates entry of peripheral cytokines and immune cells into the brain. This vascular effect amplifies neuroinflammation and oxidative damage, feeding into AD pathology [203,204].

In contrast, adiponectin exerts protective, insulin-sensitizing, and anti-inflammatory effects. Secreted by adipocytes in trimeric, hexameric, and high-molecular-weight (HMW) forms, adiponectin binds to AdipoR1 and AdipoR2 receptors present on neurons, astrocytes, oligodendrocytes, and endothelial cells [205,206]. Activation of these receptors stimulates AMPK and PPAR-α, enhancing glucose uptake via GLUT3, promoting fatty acid oxidation, improving mitochondrial biogenesis through PGC1α, and inhibiting NF-κB–mediated cytokine production [179,197,207,208]. Adiponectin also activates ERK1/2, contributing to neurotrophic signaling and synaptic maintenance [180]. In experimental AD models, exogenous adiponectin or AdipoR agonists yield striking benefits: improved spatial memory, increased hippocampal synaptic density, reduced Aβ deposition and tau phosphorylation, and lower markers of neuroinflammation and oxidative damage [209]. Notably, adiponectin-deficient mice exhibit accelerated neurodegeneration with earlier onset of cognitive deficits, increased Aβ, and elevated GSK-3β activity, reinforcing adiponectin’s neuroprotective role [210,211]. Clinical studies examining adiponectin levels in AD have yielded conflicting results. Lower adiponectin concentrations are often observed in preclinical Alzheimer’s or mild cognitive impairment (MCI), aligning with its protective functions. However, elevated adiponectin in established AD has also been reported, suggesting a compensatory increase or “adiponectin resistance” where receptor signaling is impaired despite ligand abundance, mirroring patterns seen with leptin in obesity [212,213]. Understanding these dynamics across disease stages is critical for interpreting adiponectin as a biomarker or therapeutic target (Table 3).

In terms of biomarkers, combined measurements of resistin and adiponectin in blood and CSF may yield improved early risk stratification models. Adiponectin/resistin ratios correlate with insulin sensitivity, systemic inflammation, and cognitive decline. Integrating these adipokine measures with neuroimaging (FDG–PET, volumetric MRI) and APOE-genotyping may refine predictive algorithms for AD risk and progression [224,225]. However, achieving clinical utility requires standardized assays, longitudinal validation, and adjustment for potential confounders such as age, sex, BMI, and metabolic comorbidities. Looking ahead, precision-based approaches targeting adipokines hold promise [224,225]. Personalized interventions might use genetics and metabolic profiling to identify individuals who would benefit most from adiponectin stimulation or resistin inhibition. Innovative delivery methods, such as intranasal AdipoR agonists or targeted brain delivery via viral vectors, may bypass peripheral resistance and enhance central effects with fewer systemic side effects (Table 3).

Key points, Resistin and Adiponectin in AD:

1. Resistin promotes NF-κB–dependent inflammation, insulin resistance, BBB disruption, and aggravation of Aβ/tau pathology. These actions accelerate synaptic dysfunction and cerebral metabolic failure.
2. Adiponectin counters inflammation and insulin resistance; higher functional adiponectin is associated with neuroprotection. Stage-dependent “adiponectin resistance” may blunt these benefits in AD.
3. The resistin–adiponectin balance/ratio reflects metabolic–neuroinflammatory risk and has biomarker potential.
4. Therapeutic angle: boost adiponectin signaling and/or inhibit resistin pathways within precision, stage-aware interventions.

## 8. Natural Compounds Targeting Adipokines Signaling in Alzheimer’s Disease in the Context of Obesity

Over the past two decades, natural compounds derived from plants, marine organisms, and dietary sources have received increasing attention as modulators of metabolic and neuroinflammatory pathways implicated in AD [226,227]. Among their diverse molecular targets, adipokine signaling represents a particularly promising axis. Dysregulation of adiponectin, resistin, and leptin in obesity and AD establishes a pro-inflammatory and metabolically impaired environment in the brain. While synthetic drugs such as thiazolidinediones or selective receptor agonists have been evaluated, natural compounds offer pleiotropic benefits with relatively low toxicity and long histories of human consumption [228]. Importantly, many phytochemicals restore insulin sensitivity, attenuate TLR4 signaling, enhance AMPK activity, or modulate PPARs, mechanisms directly linked to adipokine biology [229]. Recent studies highlight polyphenols as major contributors to this process. Quercetin, a flavonol abundant in apples and onions, enhances adiponectin expression through AMPK activation and PPARγ modulation in adipocytes, leading to improved systemic insulin sensitivity [230]. In transgenic AD mice, quercetin reduces amyloid deposition and rescues synaptic plasticity, effects partly mediated by increased adiponectin receptor (AdipoR1/2) expression in hippocampal neurons and astrocytes [231,232]. Similarly, epigallocatechin gallate (EGCG), the major catechin in green tea, attenuates resistin-induced TLR4/NF-κB signaling, thereby suppressing microglial activation [233]. EGCG also upregulates adiponectin in adipose tissue and enhances glucose utilization in neurons, suggesting dual systemic and central benefits [234,235]. Resveratrol, a stilbene found in grapes and red wine, stimulates SIRT1 and AMPK signaling, both downstream of adiponectin, and increases circulating adiponectin in obese rodents. In APP/PS1 mice, resveratrol improves memory performance, reduces Aβ burden, and attenuates tau phosphorylation. Notably, clinical trials with resveratrol have shown mixed results, possibly due to poor bioavailability; however, adiponectin modulation remains a consistent biomarker of its metabolic actions [236,237,238]. Curcumin, the principal polyphenol in turmeric, enhances adiponectin secretion and suppresses resistin expression in obese rodents. In neuronal contexts, curcumin decreases BACE1 activity and downregulates pro-inflammatory cytokines in microglia. While curcumin’s effects are multifaceted, its ability to shift the adipokine profile toward an anti-inflammatory balance may contribute substantially to its neuroprotective capacity. Collectively, polyphenols exert adipokine-mediated benefits through stimulation of adiponectin release and receptor activation, inhibition of resistin-driven TLR4/NF-κB cascades, and improvement of neuronal glucose metabolism via AMPK signaling, supporting their potential as dietary interventions for obesity-associated AD [239,240,241].

Beyond polyphenols, terpenoids and related compounds also show activity on adipokine pathways. Ginsenoside Rb1, isolated from Panax *ginseng*, upregulates adiponectin and PPARγ in adipocytes while reducing resistin secretion. In AD mice, Rb1 improves spatial memory and reduces hippocampal inflammation. Asiatic acid, a pentacyclic triterpene from *Centella asiatica*, enhances adiponectin signaling and reduces oxidative stress markers in cortical neurons [242,243,244]. Carotenoids such as astaxanthin and lycopene also modulate adipokines. Astaxanthin increases plasma adiponectin and decreases resistin in obese models, while reducing neuroinflammatory markers in hippocampal tissue [245,246,247]. These terpenoids highlight the capacity of natural compounds to simultaneously target peripheral adipokine secretion and central neuroinflammation, with lipophilic properties favoring blood–brain barrier penetration and enhancing translational potential. Dietary lipids such as omega-3 fatty acids further illustrate this mechanism. Eicosapentaenoic acid (EPA) and docosahexaenoic acid (DHA) consistently increase adiponectin levels in both animal and human studies. In AD models, DHA supplementation improves synaptic integrity, reduces Aβ deposition, and enhances cognitive performance. Mechanistically, omega-3 fatty acids attenuate resistin expression in macrophages and inhibit TLR4-mediated inflammatory signaling. Conjugated linoleic acid (CLA) supplementation also enhances adiponectin expression, decreases resistin, and improves systemic insulin sensitivity [248,249]. Although evidence in AD remains limited, these findings support lipid-based strategies as promising adipokine modulators.

Additional natural compounds include several alkaloids. Berberine, an isoquinoline alkaloid from *Berberis* species, increases adiponectin levels and decreases resistin in insulin-resistant rodents. Berberine also activates AMPK in neurons, improving glucose uptake and reducing oxidative stress [250,251]. Capsaicin, the pungent component of chili peppers, enhances adiponectin secretion and improves hippocampal plasticity in obese models [252,253]. These diverse compounds highlight that multiple phytochemical classes converge on the adipokine axis. Mechanistically, their effects intersect with key AD pathologies. By boosting adiponectin and suppressing resistin, polyphenols, terpenoids, and bioactive lipids enhance AMPK and PPARα signaling in neurons, improving mitochondrial biogenesis and synaptic energy supply. Simultaneously, they inhibit TLR4/NF-κB activation, reducing microglial-driven neuroinflammation. Furthermore, adipokine modulation indirectly influences amyloid and tau pathology: adiponectin activation reduces BACE1 expression and tau kinase activity, while resistin suppression prevents upregulation of GSK3β. Together, these mechanisms support a more resilient neuro-metabolic environment. Despite compelling preclinical evidence, clinical translation remains limited. Human trials of resveratrol, curcumin, and omega-3s show modest cognitive benefits, but adipokine modulation is rarely measured as a primary endpoint. Inter-individual variability, bioavailability challenges, and differences in obesity phenotypes complicate interpretation. Nanoparticle-based delivery systems and intranasal formulations are being developed to overcome these limitations [254,255]. Furthermore, combinatorial approaches integrating natural compounds with conventional anti-amyloid or anti-diabetic therapies may offer synergistic benefits. In conclusion, natural compounds targeting adipokine signaling offer a unique opportunity to address the metabolic–inflammation axis linking obesity and AD. By restoring adiponectin activity, suppressing resistin-driven inflammation, and overcoming leptin resistance, these agents support both systemic and central resilience. However, rigorous human trials are urgently needed to validate adipokines as mechanistic biomarkers of natural compound efficacy. Future research should integrate adipokine endpoints into clinical trials, stratify participants by metabolic status, and optimize delivery systems to ensure central bioavailability. Given the global rise in obesity and AD, harnessing natural compounds to modulate adipokines represents a promising and multifaceted strategy for prevention and therapy.

Key points for Natural Compounds and Adipokines in AD:

1. Phytochemicals shift the adipokine axis toward higher adiponectin and lower resistin activity.
2. Mechanistically: activate AMPK/PPAR signaling and suppress TLR4–NF-κB pathways, improving insulin sensitivity and neuronal energetics while dampening neuroinflammation. These effects can indirectly reduce Aβ/tau stress and support synaptic function.
3. Evidence is preclinically strong but mixed in humans. Next steps: stage-/metabolic-stratified trials with adipokine endpoints and improved bioavailability/delivery strategies.

## 9. Therapeutic Modulation of Adipokines in AD: Antidiabetic Agents and Natural Compounds

Antidiabetic agents impacting adipokines and cognition, Glucagon-like peptide-1 receptor agonists reduce systemic inflammation, tend to increase adiponectin and lower resistin, and may improve central insulin signaling [256]. Evidence from metabolic and early cognitive studies suggests potential benefits on memory and neuroinflammation, although weight loss and improved vascular–metabolic status are important confounders [257]. Safety in older adults requires monitoring for excessive weight loss, dehydration, and gastrointestinal symptoms. Metformin activates AMPK, improves insulin sensitivity, and may modulate neuroinflammation and adult neurogenesis [258]. Epidemiological signals are mixed, possibly due to confounding by indication and heterogeneity in exposure duration and B12 status; routine monitoring of vitamin B12 is advisable in older adults. Thiazolidinediones (e.g., pioglitazone) activate PPARγ, robustly increase adiponectin, and exert anti-inflammatory actions; mixed cognitive outcomes likely reflect dose, duration, and patient selection [259,260]. Edema and heart-failure risk must be weighed in frail patients. SGLT2 inhibitors show systemic anti-inflammatory and hemodynamic effects and may shift brain fuel utilization; direct evidence in AD is still limited. DPP-4 inhibitors indirectly enhance incretin tone and can lower pro-inflammatory cytokines; cognitive data remain preliminary.

Intranasal insulin has shown modest, short-term cognitive benefits in people with amnestic MCI and mild AD, with response heterogeneity linked to patient biology. Acute studies using regular human insulin report improvements in episodic and working memory, without measurable changes in peripheral glucose or insulin [261]. By contrast, small trials with insulin detemir suggest benefits in ε4 carriers, highlighting that genotype and formulation may shape efficacy [262]. Through studies, treatment is generally well tolerated, with mostly mild local adverse events and no meaningful hypoglycemia. Overall, effects appear transient and variable, implying that sustained benefit will likely require careful patient stratification and optimization of dose, formulation, and delivery device.

In the past year, multiple studies have reported that natural compounds modulate adipokine levels and are associated with effects on brain function (Table 4). Nonetheless, most of these observations require confirmation in well-powered clinical trials to determine their true impact on humans. For example, Resveratrol activates SIRT1/AMPK, can increase adiponectin and reduce inflammatory signaling; oral bioavailability is low, formulations and dosing schedules matter [263]. Curcumin down-modulates NF-κB and pro-inflammatory adipokines; limited brain penetration and rapid metabolism necessitate enhanced-bioavailability formulations [255]. Berberine improves insulin sensitivity via AMPK and may reduce resistin; watch for GI intolerance and drug interactions (CYPs/P-gp) [250]. Quercetin exhibits anti-inflammatory and potential anti-amyloid actions; tolerability and liver safety at higher doses require attention [230]. Overall, variability in preparations, doses, and endpoints explains inconsistent results; standardization and head-to-head trials are needed.

Key points for Therapeutic Modulation of Adipokines in AD:

1. Antidiabetic agents slope the adipokine/insulin axis.
2. Cognitive benefits to date are modest/heterogeneous and often confounded by weight loss and vascular–metabolic improvements.
3. Intranasal insulin yields short-term memory gains with genotype/formulation-dependent responses.
4. Trial priorities: patient stratification (stage/metabolic profile), rigorous safety monitoring, and standardized outcomes anchored to adipokine and insulin-signaling endpoints.

## 10. Future Directions

Obesity has emerged as a critical driver of AD progression, not only through its well-known metabolic consequences but also via adipose tissue-derived hormones, adipokines, that directly influence the brain’s pathological cascades. While rodent and in vitro studies consistently illustrate that adipokines such as leptin, adiponectin, and resistin modulate Aβ and tau accumulation, enhance or impair neuroinflammation, and affect neuronal metabolism, translation to human data remains inconsistent. Several cohort studies highlight significant divergences between preclinical mechanistic clarity and the complexity of human physiology, revealing crucial gaps that must be addressed if adipokine-centered therapies are to be realized in clinical practice [191,274].

Obesity induces chronic low-grade systemic inflammation, insulin resistance, and dysregulated adipokine secretion, creating a milieu that promotes neurodegeneration. Adipokines cross the BBB or act peripherally to prime microglia, disrupt neuronal signaling, and alter proteinopathy dynamics. In animal models, adiponectin enhances AMPK and PPAR-α signaling, mitigating Aβ aggregation and tau phosphorylation, whereas resistin triggers neuroinflammation via TLR4/NF-κB pathways, fostering amyloidogenesis. Leptin, under physiological low-fat conditions, activates hippocampal PI3K-Akt and MAPK cascades to support synaptic plasticity and reduce amyloidogenic processing. However, in obesity, leptin resistance, marked by elevated circulating leptin but impaired BBB transport and receptor desensitization, blunts this neuroprotective axis, accelerating both amyloid and tau pathology. Rodent and cell models offer strong mechanistic proof: adiponectin administration lowers Aβ42 burden in AD mice, improves cognition, and partially restores glucose metabolism; resistin increases pro-inflammatory cytokines and small Aβ aggregates; leptin reduces Aβ plaques and tau phosphorylation while enhancing synaptic function, effects that are significantly blunted by obesity-induced dysfunction. These models inform potential therapeutic pathways, such as selective AdipoR1/R2 agonists or resistin inhibitors. However, human data present a more complex picture [144,274].

Several human cohort studies yield contradictory adipokine associations with AD. Has been described that leptin’s protective correlation against cognitive decline is observed in non-obese individuals but lost in overweight ones, reflecting midlife leptin resistance in obesity. JAMA Network data show plasma leptin inversely correlates with amyloid and tau PET load in older adults, yet this protective signal diminishes when leptin resistance confounds central action [275,276]. Similarly, resistin is elevated in obese individuals but clinical associations with dementia differ depending on age, obesity phenotypes, and comorbidities, reflecting uncertainties around human secretory dynamics. Adiponectin, though decreased in obesity and promising in preclinical studies as insulin-sensitizing and anti-inflammatory, shows inconsistent links with human cognitive decline, variably associated with both protective and risk profiles across studies. These human–animal discrepancies may stem from: (1) species differences in adipokine expression and function, e.g., resistin originates from adipocytes in rodents but from immune cells in humans; (2) variability in adipose distribution (visceral vs. subcutaneous) and differing BBB integrity in obesity, altering transport; (3) genetic and epigenetic diversity; (4) coexisting comorbidities such as type 2 diabetes mellitus, cardiovascular disease, and depression; and (5) sex and hormonal modulations, particularly postmenopausal estrogen loss enhancing resistin and lowering adiponectin, worsening adipokine balance [277,278,279].

Notably, in older adults and preclinical dementia states, higher adiponectin may track with weight loss and frailty rather than protection, a phenomenon likely reflecting reverse causation and survival bias. This underscores the need to interpret adipokine levels considering longitudinal weight trajectories and inflammatory burden. Furthermore, cohort studies show paradoxical findings on weight: mid-life obesity increases AD risk, but unintentional late-life weight loss often precedes clinical dementia, complicating the interpretation of adipokine levels. This temporal variability underscores that a single adipokine measurement may not reflect dynamic central signaling [280]. Given these discrepancies, more focused human-relevant models are essential. iPSC-derived neurons, brain organoids, or humanized mouse models could capture adipokine signaling within human-like neuronal and glial environments. Clinical studies must stratify participants by obesity phenotypes, leptin resistance status, metabolic comorbidities, and genetic risk factors such as APOE-ε4 to assess context-dependent adipokine effects. Therapeutically, weight-loss interventions, insulin-sensitizers, and GLP-1 agonists can improve adipokine profiles but lack specificity. Novel targeted agents, like adiponectin receptor agonists (e.g., AdipoRon) or resistin pathway inhibitors, demonstrate benefits in preclinical models, restoring central insulin sensitivity and dampening neuroinflammation [280]. However, human trials are lacking, and optimal delivery modalities, such as intranasal systems, nanoparticles, or exosome carriers, must be explored to bypass systemic resistance and focus action within the CNS.

Longitudinal, well-powered clinical trials are needed to correlate peripheral adipokine levels, central CSF or PET biomarkers of Aβ and tau, and cognitive endpoints. These studies should include obese and non-obese individuals across age spans to determine whether adipokine modulation can delay progression from metabolic dysfunction or MCI to AD. Additional readouts, such as fluid biomarkers (neurofilament light, inflammatory markers), neuroimaging, and adipokine gene expression, will enrich mechanistic understanding. Precision medicine frameworks leveraging adipokine biology represent an important frontier. Genetic polymorphisms in related adiponectin, leptin, or resistin signaling genes may predict responsiveness to adipokine-targeted therapies. AI-driven stratification models integrating metabolic, genetic, imaging, and biochemical data could identify subsets likely to benefit. Sex-specific effects must also be integrated: postmenopausal women with obesity may be particularly vulnerable due to adipose and inflammatory shifts affecting leptin and resistin. Interventions tailored to hormonal profiles may yield better outcomes. Public health strategies are similarly relevant. With rising global obesity rates, especially in aging populations, recognizing adipokines as mediators of AD risk could inform screening programs. Simple assays of leptin/adiponectin ratios, resistin levels, or composite adipokine panels might identify individuals at metabolic risk for cognitive decline. Educational campaigns linking metabolic and cognitive health could encourage earlier lifestyle interventions. Also, systems biology technologies, such as single-cell transcriptomics, spatial proteomics, and CRISPR models, are crucial to dissect cell-type–specific adipokine action in human brain tissue. Gut microbiota–adipokine–brain axes also offer promising insights, as obesity-driven microbiome changes influence adipokine production and may exacerbate neurodegenerative processes.

Key points for Future Directions:

1. Strong preclinical effects of leptin/adiponectin/resistin on Aβ/tau, neuroinflammation, and metabolism do not consistently replicate in humans due to species, obesity phenotype, leptin resistance, comorbidities, and sex/hormonal factors.
2. Run longitudinal, powered studies linking peripheral adipokines with CSF/PET Aβ/tau, imaging, fluid biomarkers, and cognition, stratified by obesity phenotype, leptin resistance, APOE, and age.
3. Use genetics, multimodal AI models, and sex-specific approaches; evaluate simple screening panels and integrate microbiome and single-cell/spatial omics to map cell-specific actions.

## 11. General Conclusions

The convergence of obesity and AD highlights adipokines as critical mediators linking metabolic dysfunction, inflammation, and neurodegeneration. Among them, adiponectin, resistin, and leptin play opposing roles in shaping brain resilience and vulnerability, positioning adipokine signaling at the forefront of therapeutic innovation.

Preclinical studies provide strong evidence that adipokines modulate insulin sensitivity, neuroinflammation, and amyloid/tau pathology. However, translation to humans remains inconsistent, with contradictory findings across observational studies. These discrepancies, likely influenced by sex, age, ethnicity, genetic background, and comorbidities, underscore the urgent need for rigorous, longitudinal, and stratified human studies. Bridging this gap is fundamental if adipokine-targeted strategies are to evolve into effective and safe treatments. From a therapeutic standpoint, adipokines embody the themes of this Special Issue by intersecting emerging pharmacological strategies and natural compounds. AdipoR agonists, resistin inhibitors, and advanced delivery systems illustrate the potential of precision pharmacology, while phytochemicals such as quercetin, EGCG, resveratrol, and curcumin demonstrate the capability of multi-target metabolic modulators. Equally relevant are lifestyle-based approaches, exercise, caloric restriction, and dietary interventions, which modulate adipokine balance and may synergize with pharmacological therapies.

Ultimately, adipokines hold dual value as both biomarkers and therapeutic targets, offering opportunities for early detection, patient stratification, and combinatorial approaches that integrate metabolic and cognitive benefits. Moving forward, well-designed human studies are indispensable to validate preclinical findings and resolve current inconsistencies. Only by strengthening this translational bridge will it be possible to harness adipokine biology for the development of effective therapies against obesity-associated AD, aligning with the mission to highlight innovative and sustainable treatment paradigms. Clinically, integrating simple metabolic panels with adipokine profiling may help identify patients most likely to benefit from combined lifestyle, antidiabetic, and nutraceutical strategies. Prospective, well-phenotyped trials that co-track adipokines, insulin resistance, vascular function, and cognition are now essential to clarify who benefits, from what, and when.

Key points for General Conclusions:

1. Closing the translational gap: There is strong preclinical evidence that adipokines shape insulin signaling, neuroinflammation, and Aβ/tau which does not translate consistently to humans, demanding rigorous, longitudinal, stratified trials to define who benefits, from what, and when.
2. Dual clinical utility: Adipokines are both biomarkers and targets; integrating adipokine profiling with precision therapeutics (AdipoR agonists, resistin inhibitors, advanced delivery) plus lifestyle/nutraceutical strategies can enable early detection, patient stratification, and combinatorial treatment.

## Figures and Tables

**Table 1:** Adipokines are most studied for brain function.

Adipokine	Main Cerebral Effect	Experimental Model	Reference
Leptin	Enhances synaptic plasticity (LTP), neurogenesis; reduces Aβ	Transgenic mice (db/db, APP/PS1), hippocampal slices, neuronal cultures	[141]
Adiponectin	Anti-inflammatory, neuroprotective, promotes neurogenesis; crosses BBB	Obese and ischemic mice, CSF from humans, neuronal and glial cultures	[142]
Visfatin (NAMPT)	Enhances BDNF, neuronal survival, modulates neuroinflammation	High-fat diet (HFD) mice, glial and hippocampal cultures, inflammatory models	[143]
Resistin	Increases IL-6 and TNF-α; promotes neuroinflammation	Obese mice, Alzheimer’s models, clinical plasma/CSF studies	[144]
Chemerin	Modulates neuroinflammation and oxidative stress	HFD mice, endothelial cultures; CMKLR1 expression in brain	[145,146]
Apelin	Neurotrophic effects; improves feeding behavior and neurovascular function	Obese mice, neuronal cultures, intracerebral injection studies	[147]
Omentin-1	Anti-inflammatory, potential neuroprotective action	HFD and insulin-resistant models; indirect brain associations	[148]
PAI-1	Disrupts BBB integrity; contributes to neurodegeneration	Stroke models in mice, endothelial BBB models	[149]
RBP4	Associated with cognitive decline, affects hippocampal function	Clinical obesity/cognition studies; in vitro BBB and neuronal assays	[150]
Vaspin	Anti-inflammatory effects, possible neuroprotection	HFD mice, glial cultures (brain-specific evidence limited)	[151]
Progranulin	Modulates microglial activity, neuroinflammatory regulation	ALS and EAE models, human CSF analysis	[152]

**Table 2:** Levels of adipokines described in human studies.

Adipokine	Sample and Population	Main Findings in Elderly	Observed Discordance	Reference
Adiponectin	Plasma (older adults with MCI, AD, depression)	Low levels associated with MCI and inflammation- High levels in Aβ individuals predict cortical thinning	Appears neuroprotective in some studies, yet high levels predict atrophy in Aβ MCI	[191,192]
Leptin	Plasma and CSF (cognitively healthy, MCI, AD)	Lower plasma leptin linked to lower CSF Aβ and worse cognition- Some studies find no clear association	Inconsistent link between plasma/CSF leptin and cognitive outcomes or biomarkers	[161,193]
Visfatin	Plasma (elderly with obesity or diabetes)	Suggested neuroprotective and inflammatory roles, but inconsistent concentrations across cohorts	No consensus on whether it rises or falls in aging; mechanisms remain unclear	[194]
Adipsin	Plasma (early AD cohorts, middle-aged and older adults)	Proposed as early biomarker with leptin and adiponectin, but limited age-specific trend data	Preliminary findings; lack of consistent replication in large elderly cohorts	[195,196]

**Table 3:** Comparative Profile of Adiponectin and Resistin in Obesity-Associated Alzheimer’s Disease (↑: increase; ↓: decrease).

Feature	Adiponectin	Resistin	References
Primary Source	Adipocytes (mainly subcutaneous WAT)	Macrophages and mononuclear immune cells (in humans)	[19]
Levels in Obesity	↓ Decreased	↑ Increased	[214,215]
Receptors in Brain	AdipoR1 (neurons), AdipoR2 (astrocytes, endothelial cells)	TLR4, CAP1 (microglia, astrocytes)	[216,217]
Main CNS Effects	↑ Glucose uptake, ↓ ROS, ↑ mitochondrial biogenesis, ↓ neuroinflammation, ↑ synaptic plasticity	↑ NF-κB activation, ↑ ROS, ↑ cytokine release, ↓ insulin signaling, ↑ BACE1 and tau phosphorylation	[218,219]
Mechanistic Pathways	AMPK, PPARα, ERK1/2, PGC1α	TLR4–NF-κB, ERK1/2, GSK3β	[217,220]
Impact on Aβ and Tau	↓ Aβ deposition, ↓ tau phosphorylation	↑ Aβ generation (↑ BACE1), ↑ tau phosphorylation (↑ GSK3β)	[144,211]
Effect on BBB Integrity	Preserves BBB (↑ eNOS, ↓ endothelial inflammation)	Disrupts BBB (↓ tight junctions, ↑ MMP activity)	[174,221]
Observed in AD Models	Improves memory, reduces pathology	Correlates with cognitive decline, hippocampal atrophy	[144,222]
Therapeutic Strategies	AdipoR agonists (AdipoRon), PPAR-γ agonists (pioglitazone), AMPK activators (metformin)	TLR4 antagonists, CAP1 inhibitors, siRNA/antisense therapies	[217,223]

**Table 4:** Natural compounds that modulate adipokines and brain outcomes in Alzheimer’s disease. (↑: increase; ↓: decrease).

Compounds	Primary Adipokine Effect	Reported Brain Effect	Reference
Resveratrol (polyphenol)	↑ Adiponectin; ↓ inflammatory tone	↓ Aβ/tau stress; ↓ microglial activation; improved memory and glucose metabolism in models	[264,265]
Curcumin (polyphenol)	↑ Adiponectin; ↓ Resistin (preclinical)	↓ Neuroinflammation; ↓ Aβ; support synapses in models	[266]
Quercetin (flavonol)	↑ Adiponectin signaling (AdipoR↑)	↓ Aβ deposition; ↑ synaptic plasticity; antioxidant neuroprotection	[267]
Berberine (alkaloid)	↑ Adiponectin; ↓ Resistin	↑ Glucose uptake; ↓ oxidative stress; cognitive benefit in models	[268,269]
Astaxanthin (carotenoid)	↑ Adiponectin; ↓ Resistin (models)	↑ Aβ clearance; ↓ BBB inflammation; memory benefit (models)	[270,271]
Omega-3 PUFAs (EPA/DHA)	↑ Adiponectin; ↓ Resistin (immune cells)	↓ Aβ; ↑ synaptic integrity; better cognition (models); mixed human cognition	[272]
Capsaicin (vanilloid)	↑ Adiponectin secretion	↓ Aβ generation; improved plasticity in models	[253,273]

## Data Availability

No new data were created or analyzed in this study. Data sharing is not applicable to this article.

## References

[1] Kelly T., Yang W., Chen C.S., Reynolds K., He J. (2008). Global burden of obesity in 2005 and projections to 2030. Int. J. Obes..

[2] Scheen A.J., Paquot N. (2015). Obesity: A new paradigm for treating obesity and diabetes mellitus. Nat. Rev. Endocrinol..

[3] Despres J.P., Arsenault B.J., Cote M., Cartier A., Lemieux I. (2008). Abdominal obesity: The cholesterol of the 21st century?. Can. J. Cardiol..

[4] Naderali E.K., Ratcliffe S.H., Dale M.C. (2009). Obesity and alzheimer’s disease: A link between body weight and cognitive function in old age. Am. J. Alzheimer’s Dis. Other Dement..

[5] Pugazhenthi S., Qin L., Reddy P.H. (2017). Common neurodegenerative pathways in obesity, diabetes, and Alzheimer’s disease. Biochim. Biophys. Acta Mol. Basis Dis..

[6] Ly M., Yu G.Z., Mian A., Cramer A., Meysami S., Merrill D.A., Samara A., Eisenstein S.A., Hershey T., Babulal G.M. (2023). Neuroinflammation: A Modifiable Pathway Linking Obesity, Alzheimer’s disease, and Depression. Am. J. Geriatr. Psychiatry.

[7] Profenno L.A., Porsteinsson A.P., Faraone S.V. (2010). Meta-Analysis of Alzheimer’s Disease Risk with Obesity, Diabetes, and Related Disorders. Biol. Psychiatry.

[8] Korczyn A.D., Grinberg L.T. (2024). Is Alzheimer disease a disease?. Nat. Rev. Neurol..

[9] Querfurth H.W., LaFerla F.M. (2010). Alzheimer’s disease. N. Engl. J. Med..

[10] Serrano-Pozo A., Frosch M.P., Masliah E., Hyman B.T. (2011). Neuropathological alterations in Alzheimer disease. Cold Spring Harb. Perspect. Med..

[11] Rios J.A., Cisternas P., Arrese M., Barja S., Inestrosa N.C., Ríos Ja Cisternas P., Arrese M., Barja S., Inestrosa N.C. (2014). Is Alzheimer’s disease related to metabolic syndrome? A Wnt signaling conundrum. Prog. Neurobiol..

[12] Lv Y., Xiang Y., Fu W., Li B., Li X. (2025). Global burden trends of Alzheimer’s disease and other dementias attributable to smoking from 1990 to 2021. J. Alzheimer’s Dis..

[13] Makin S. (2025). The future of Alzheimer’s treatment. Nature.

[14] Long J.M., Holtzman D.M. (2019). Alzheimer Disease: An Update on Pathobiology and Treatment Strategies. Cell.

[15] Morris J.K., Burns J.M. (2012). Insulin: An emerging treatment for Alzheimer’s disease dementia?. Curr. Neurol. Neurosci. Rep..

[16] Businaro R., Ippoliti F., Ricci S., Canitano N., Fuso A. (2012). Alzheimer’s disease promotion by obesity: Induced mechanisms-molecular links and perspectives. Curr. Gerontol. Geriatr. Res..

[17] Craft S. (2005). Insulin resistance syndrome and Alzheimer’s disease: Age- and obesity-related effects on memory, amyloid, and inflammation. Neurobiol. Aging.

[18] Mandrekar S., Landreth G.E. (2010). Microglia and Inflammation in Alzheimers Disease. CNS Neurol. Disord. Drug Targets.

[19] Kiliaan A.J., Arnoldussen I.A.C., Gustafson D.R. (2014). Adipokines: A link between obesity and dementia?. Lancet Neurol..

[20] Ahima R.S., Osei S.Y. (2008). Adipokines in obesity. Front. Horm. Res..

[21] Aguilar-Valles A., Inoue W., Rummel C., Luheshi G.N. (2015). Obesity, adipokines and neuroinflammation. Neuropharmacology.

[22] Quan N., Banks W.A. (2007). Brain-immune communication pathways. Brain Behav. Immun..

[23] Brookmeyer R., Johnson E., Ziegler-Graham K., Arrighi H.M. (2007). Forecasting the global burden of Alzheimer’s disease. Alzheimer’s Dement..

[24] Nichols E., Szoeke C.E.I., Vollset S.E., Abbasi N., Abd-Allah F., Abdela J., Aichour M.T.E., Akinyemi R.O., Alahdab F., Asgedom S.W. (2019). Global, regional, and national burden of Alzheimer’s disease and other dementias, 1990–2016: A systematic analysis for the Global Burden of Disease Study 2016. Lancet Neurol..

[25] He D., Liu M., Tang Y., Tian X., Zhou L., Chen Y., Liu X. (2025). Systematic analysis and prediction of the burden of Alzheimer’s disease and other dementias caused by hyperglycemia. Front. Public Health.

[26] Mallapaty S. (2025). One potent gene raises risk of Alzheimer’s, Parkinson’s and other brain diseases. Nature.

[27] Jackson K., Barisone G.A., Diaz E., Jin L.W., DeCarli C., Despa F. (2013). Amylin deposition in the brain: A second amyloid in Alzheimer disease?. Ann. Neurol..

[28] Tai L.M., Ghura S., Koster K.P., Liakaite V., Maienschein-Cline M., Kanabar P., Collins N., Ben-Aissa M., Lei A.Z., Bahroos N. (2015). APOE-modulated Aβ-induced neuroinflammation in Alzheimer’s disease: Current landscape, novel data, and future perspective. J. Neurochem..

[29] Jackson R.J., Hyman B.T., Serrano-Pozo A. (2024). Multifaceted roles of APOE in Alzheimer disease. Nat. Rev. Neurol..

[30] Bertram L., Lange C., Mullin K., Parkinson M., Hsiao M., Hogan M.F., Schjeide B.M., Hooli B., Divito J., Ionita I. (2008). Genome-wide association analysis reveals putative Alzheimer’s disease susceptibility loci in addition to APOE. Am. J. Hum. Genet..

[31] Cisternas P., Inestrosa N.C. (2017). Brain glucose metabolism: Role of Wnt signaling in the metabolic impairment in Alzheimer’s disease. Neurosci. Biobehav. Rev..

[32] Liu P., Wang Z.H., Kang S.S., Liu X., Xia Y., Chan C.B., Ye K. (2022). High-fat diet-induced diabetes couples to Alzheimer’s disease through inflammation-activated C/EBPβ/AEP pathway. Mol. Psychiatry.

[33] Newcombe E.A., Camats-Perna J., Silva M.L., Valmas N., Huat T.J., Medeiros R. (2018). Inflammation: The link between comorbidities, genetics, and Alzheimer’s disease. J. Neuroinflamm..

[34] Godoy J.A., Rios J.A., Zolezzi J.M., Braidy N., Inestrosa N.C. (2014). Signaling pathway cross talk in Alzheimer’s disease. Cell Commun. Signal..

[35] Attwell D., Laughlin S.B. (2001). An energy budget for signaling in the grey matter of the brain. J. Cereb. Blood Flow Metab..

[36] Zhang X., Alshakhshir N., Zhao L. (2021). Glycolytic Metabolism, Brain Resilience, and Alzheimer’s Disease. Front. Neurosci..

[37] Cunnane S., Nugent S., Roy M., Courchesne-Loyer A., Croteau E., Tremblay S., Castellano A., Pifferi F., Bocti C., Paquet N. (2011). Brain fuel metabolism, aging, and Alzheimer’s disease. Nutrition.

[38] Marcus C., Mena E., Subramaniam R.M. (2014). Brain PET in the diagnosis of Alzheimer’s disease. Clin. Nucl. Med..

[39] Talbot K., Wang H.Y., Kazi H., Han L.Y., Bakshi K.P., Stucky A., Fuino R.L., Kawaguchi K.R., Samoyedny A.J., Wilson R.S. (2012). Demonstrated brain insulin resistance in Alzheimer’s disease patients is associated with IGF-1 resistance, IRS-1 dysregulation, and cognitive decline. J. Clin. Investig..

[40] Wei Z., Koya J., Reznik S.E. (2021). Insulin Resistance Exacerbates Alzheimer Disease via Multiple Mechanisms. Front. Neurosci..

[41] Arnold S.E., Arvanitakis Z., Macauley-Rambach S.L., Koenig A.M., Wang H.Y., Ahima R.S., Craft S., Gandy S., Buettner C., Stoeckel L.E. (2018). Brain insulin resistance in type 2 diabetes and Alzheimer disease: Concepts and conundrums. Nat. Rev. Neurol..

[42] Mosconi L. (2013). Glucose metabolism in normal aging and Alzheimer’s disease: Methodological and physiological considerations for PET studies. Clin Transl Imaging.

[43] Mosconi L. (2005). Brain glucose metabolism in the early and specific diagnosis of Alzheimer’s disease: FDG-PET studies in MCI and AD. Eur. J. Nucl. Med. Mol. Imaging.

[44] Xu S., Lu F., Gao J., Yuan Y. (2024). Inflammation-mediated metabolic regulation in adipose tissue. Obes. Rev..

[45] Zhao J., Zhao D., Wang J., Luo X., Guo R. (2022). Inflammation—Cause or consequence of late onset Alzheimer’s disease or both? A review of the evidence. Eur. J. Inflamm..

[46] Novoa C., Salazar P., Cisternas P., Gherardelli C., Vera-Salazar R., Zolezzi J.M., Inestrosa N.C. (2022). Inflammation context in Alzheimer’s disease, a relationship intricate to define. Biol. Res..

[47] Kazemeini S., Nadeem-Tariq A., Shih R., Rafanan J., Ghani N., Vida T.A. (2024). From Plaques to Pathways in Alzheimer’s Disease: The Mitochondrial-Neurovascular-Metabolic Hypothesis. Int. J. Mol. Sci..

[48] Clark T.A., Lee H.P., Rolston R.K., Zhu X., Marlatt M.W., Castellani R.J., Nunomura A., Casadesus G., Smith M.A., Lee H.G. (2010). Oxidative Stress and its Implications for Future Treatments and Management of Alzheimer Disease. Int. J. Biomed. Sci..

[49] Yanai H., Adachi H., Hakoshima M., Katsuyama H. (2024). Pathology and Treatments of Alzheimer’s Disease Based on Considering Changes in Brain Energy Metabolism Due to Type 2 Diabetes. Molecules.

[50] Tedeschi L.G., Katz S.L. (1965). Clinicopathological Conference of the Framingham Union Hospital. BMQ.

[51] Elias M.F., Elias P.K., Sullivan L.M., Wolf P.A., D’Agostino R.B. (2005). Obesity, diabetes and cognitive deficit: The Framingham Heart Study. Neurobiol. Aging.

[52] Tan Z.S., Beiser A., Vasan R.S., Au R., Auerbach S., Kiel D.P., Wolf P.A., Seshadri S. (2008). Thyroid function and the risk of Alzheimer disease: The Framingham study. Arch. Intern. Med..

[53] Stewart R., Masaki K., Xue Q.L., Peila R., Petrovitch H., White L.R., Launer L.J. (2005). A 32-year prospective study of change in body weight and incident dementia: The Honolulu-Asia Aging Study. Arch. Neurol..

[54] Walker J.M., Harrison F.E. (2015). Shared Neuropathological Characteristics of Obesity, Type 2 Diabetes and Alzheimer’s Disease: Impacts on Cognitive Decline. Nutrients.

[55] Stickel A.M., Tarraf W., Gonzalez K.A., Isasi C.R., Kaplan R., Gallo L.C., Zeng D., Cai J., Pirzada A., Daviglus M.L. (2021). Central Obesity, Cardiometabolic Risk, and Cognitive Change in the Study of Latinos: Investigation of Neurocognitive Aging. J. Alzheimer’s Dis..

[56] Chuang Y.F., An Y., Bilgel M., Wong D.F., Troncoso J.C., O’Brien R.J., Breitner J.C., Ferruci L., Resnick S.M., Thambisetty M. (2016). Midlife adiposity predicts earlier onset of Alzheimer’s dementia, neuropathology and presymptomatic cerebral amyloid accumulation. Mol. Psychiatry.

[57] Ouchi N., Parker J.L., Lugus J.J., Walsh K. (2011). Adipokines in inflammation and metabolic disease. Nat. Rev. Immunol..

[58] Sui S.X., Pasco J.A. (2020). Obesity and brain function: The brain–body crosstalk. Medicina.

[59] Uranga R.M., Keller J.N. (2019). The complex interactions between obesity, metabolism and the brain. Front. Neurosci..

[60] Farris W., Mansourian S., Chang Y., Lindsley L., Eckman E.A., Frosch M.P., Eckman C.B., Tanzi R.E., Selkoe D.J., Gué S. (2003). Insulin-degrading enzyme regulates the levels of insulin, amyloid-protein, and the-amyloid precursor protein intracellular domain in vivo. Proc. Natl. Acad. Sci. USA.

[61] Qiu W.Q., Folstein M.F. (2006). Insulin, insulin-degrading enzyme and amyloid-β peptide in Alzheimer’s disease: Review and hypothesis. Neurobiol. Aging.

[62] Link C.D. (2007). Decreased Insulin-Receptor Signaling Promotes the Autophagic Degradation of beta-amyloid peptide in C. elegans. Autophagy.

[63] Pearson-Leary J., McNay E.C. (2012). Intrahippocampal administration of amyloid-β1-42 oligomers acutely impairs spatial working memory, insulin signaling, and hippocampal metabolism. J. Alzheimer’s Dis..

[64] Hopperton K.E., Mohammad D., Trépanier M.O., Giuliano V., Bazinet R.P. (2018). Markers of microglia in post-mortem brain samples from patients with Alzheimer’s disease: A systematic review. Mol. Psychiatry.

[65] Josephs K.A., Dickson D.W., Tosakulwong N., Weigand S.D., Murray M.E., Petrucelli L., Liesinger A.M., Senjem M.L., Spychalla A.J., Knopman D.S. (2017). Rates of hippocampal atrophy and presence of post-mortem TDP-43 in patients with Alzheimer’s disease: A longitudinal retrospective study. Lancet Neurol..

[66] Jayaraman A., Pike C.J. (2014). Alzheimer’s Disease and Type 2 Diabetes: Multiple Mechanisms Contribute to Interactions. Curr. Diabetes Rep..

[67] Kullenberg H., Rossen J., Johansson U.B., Hagströmer M., Nyström T., Kumlin M., Svedberg M.M. (2024). Correlations between insulin-degrading enzyme and metabolic markers in patients diagnosed with type 2 diabetes, Alzheimer’s disease, and healthy controls: A comparative study. Endocrine.

[68] Corraliza-Gomez M., Bermejo T., Lilue J., Rodriguez-Iglesias N., Valero J., Cozar-Castellano I., Arranz E., Sanchez D., Ganfornina M.D. (2023). Insulin-degrading enzyme (IDE) as a modulator of microglial phenotypes in the context of Alzheimer’s disease and brain aging. J. Neuroinflamm..

[69] Gong X., Liang Z., Liu W., Zhao Y., Yang Y., Wu M., Shang J., Xiao Y., Mei Y., Su Q. (2021). High Fat Diet Aggravates AD-Related Pathogenic Processes in APP/PS1 Mice. Curr. Alzheimer Res..

[70] Chen Y., Liang Z., Tian Z., Blanchard J., Dai C.L., Chalbot S., Iqbal K., Liu F., Gong C.X. (2014). Intracerebroventricular streptozotocin exacerbates Alzheimer-like changes of 3xTg-AD mice. Mol. Neurobiol..

[71] Tyagi A., Musa M., Labeikovsky W., Pugazhenthi S. (2022). Sirt3 deficiency induced down regulation of insulin degrading enzyme in comorbid Alzheimer’s disease with metabolic syndrome. Sci. Rep..

[72] Hatakawa Y., Takeuchi Y., Lee S.H., Oe T. (2024). Tyrosine modifications of insulin-degrading enzyme enable favorable control of substrate specificity for both Alzheimer’s disease and type-2 diabetes mellitus. Bioorg. Chem..

[73] Dimache A.M., Șalaru D.L., Sascău R., Stătescu C. (2021). The Role of High Triglycerides Level in Predicting Cognitive Impairment: A Review of Current Evidence. Nutrients.

[74] Mosconi L., Berti V., Glodzik L., Pupi A., De Santi S., de Leon M.J. (2010). Pre-Clinical Detection of Alzheimer’s Disease Using FDG-PET, with or without Amyloid Imaging. J. Alzheimer’s Dis..

[75] Mosconi L., Mchugh P.F. (2011). FDG-and amyloid-PET in Alzheimer’s disease: Is the whole greater than the sum of the parts?. Q. J. Nucl. Med. Mol. Imaging.

[76] Mosconi L., Tsui W.H., De Santi S., Li J., Rusinek H., Convit A., Li Y., Boppana M., de Leon M.J. (2005). Reduced hippocampal metabolism in MCI and AD: Automated FDG-PET image analysis. Neurology.

[77] Soltani S., Dolatshahi M., Soltani S., Khazaei K., Rahmani M., Raji C.A. (2025). Relationships Between Brain Glucose Metabolism Patterns and Impaired Glycemic Status: A Systematic Review of FDG-PET Studies With a Focus on Alzheimer’s Disease. Hum. Brain Mapp..

[78] Cayir S., Volpi T., Toyonaga T., Gallezot J.D., Yang Y., Sadabad F.E., Mulnix T., Mecca A.P., Fesharaki-Zadeh A., Matuskey D. (2024). Relationship between neuroimaging and cognition in frontotemporal dementia: An FDG-PET and structural MRI study. J. Neuroimaging.

[79] Liao L., Cheng D., Wang J., Duong D.M., Losik T.G., Gearing M., Rees H.D., Lah J.J., Levey A.I., Peng J. (2004). Proteomic characterization of postmortem amyloid plaques isolated by laser capture microdissection. J. Biol. Chem..

[80] Yonamine C.Y., Passarelli M., Suemoto C.K., Pasqualucci C.A., Jacob-Filho W., Alves V.A.F., Marie S.K.N., Correa-Giannella M.L., Britto L.R., Machado U.F. (2023). Postmortem Brains from Subjects with Diabetes Mellitus Display Reduced GLUT4 Expression and Soma Area in Hippocampal Neurons: Potential Involvement of Inflammation. Cells.

[81] Zhang X.X., Ma Y.H., Hu H.Y., Ma L.Z., Tan L., Yu J.T. (2022). Late-Life Obesity Associated with Tau Pathology in Cognitively Normal Individuals: The CABLE Study. J. Alzheimer’s Dis..

[82] Fasshauer M., Blüher M. (2015). Adipokines in health and disease. Trends Pharmacol. Sci..

[83] Turer A.T., Scherer P.E. (2012). Adiponectin: Mechanistic insights and clinical implications. Diabetologia.

[84] Khoramipour K., Chamari K., Hekmatikar A.A., Ziyaiyan A., Taherkhani S., Elguindy N.M., Bragazzi N.L. (2021). Adiponectin: Structure, Physiological Functions, Role in Diseases, and Effects of Nutrition. Nutrients.

[85] Steppan C.M., Brown E.J., Wright C.M., Bhat S., Banerjee R.R., Dai C.Y., Enders G.H., Silberg D.G., Wen X., Wu G.D. (2001). A family of tissue-specific resistin-like molecules. Proc. Natl. Acad. Sci. USA.

[86] Codoñer-Franch P., Alonso-Iglesias E. (2015). Resistin: Insulin resistance to malignancy. Clin. Chim. Acta.

[87] Benomar Y., Amine H., Crépin D., Al Rifai S., Riffault L., Gertler A., Taouis M. (2016). Central Resistin/TLR4 impairs adiponectin signaling, contributing to insulin and FGF21 resistance. Diabetes.

[88] Watson L.S., Wilken-Resman B., Williams A., DiLucia S., Sanchez G., McLeod T.L., Sims-Robinson C. (2022). Hyperinsulinemia alters insulin receptor presentation and internalization in brain microvascular endothelial cells. Diabetes Vasc. Dis. Res..

[89] Sripetchwandee J., Chattipakorn N., Chattipakorn S.C. (2018). Links between obesity-induced brain insulin resistance, brain mitochondrial dysfunction, and dementia. Front. Endocrinol..

[90] Huang J., Chen Y., Wang X., Wang C., Yang J., Guan B. (2023). Change in Adipokines and Gastrointestinal Hormones After Bariatric Surgery: A Meta-analysis. Obes. Surg..

[91] Becic T., Studenik C., Hoffmann G. (2018). Exercise Increases Adiponectin and Reduces Leptin Levels in Prediabetic and Diabetic Individuals: Systematic Review and Meta-Analysis of Randomized Controlled Trials. Med. Sci..

[92] Ravussin E., Redman L.M., Rochon J., Das S.K., Fontana L., Kraus W.E., Romashkan S., Williamson D.A., Meydani S.N., Villareal D.T. (2015). A 2-Year Randomized Controlled Trial of Human Caloric Restriction: Feasibility and Effects on Predictors of Health Span and Longevity. J. Gerontol. A Biol. Sci. Med. Sci..

[93] Coucha M., Abdelsaid M., Ward R., Abdul Y., Ergul A. (2018). Impact of Metabolic Diseases on Cerebral Circulation: Structural and Functional Consequences. Compr. Physiol..

[94] Jiang Q., Zhang L., Ding G., Davoodi-Bojd E., Li Q., Li L., Sadry N., Nedergaard M., Chopp M., Zhang Z. (2016). Impairment of the glymphatic system after diabetes. J. Cereb. Blood Flow Metab..

[95] Pratchayasakul W., Arunsak B., Suparan K., Sriwichaiin S., Chunchai T., Chattipakorn N., Chattipakorn S.C. (2022). Combined caloric restriction and exercise provides greater metabolic and neurocognitive benefits than either as a monotherapy in obesity with or without estrogen deprivation. J. Nutr. Biochem..

[96] Chakrabarti S. (2014). Altered serum levels of adipokines and insulin in probable Alzheimer’s disease. J. Alzheimer’s Dis..

[97] Ma J., Zhang W., Wang H.-F., Wang Z.-X., Jiang T., Tan M.-S., Yu J.-T., Tan L. (2016). Peripheral Blood Adipokines and Insulin Levels in Patients with Alzheimer’s Disease: A Replication Study and Meta-Analysis. Curr. Alzheimer Res..

[98] Xie J., Van Hoecke L., Vandenbroucke R.E. (2022). The Impact of Systemic Inflammation on Alzheimer’s Disease Pathology. Front. Immunol..

[99] Kinney J.W., Bemiller S.M., Murtishaw A.S., Leisgang A.M., Salazar A.M., Lamb B.T. (2018). Inflammation as a central mechanism in Alzheimer’s disease. Alzheimer’s Dement. Transl. Res. Clin. Interv..

[100] Griciuc A., Tanzi R.E. (2021). The role of innate immune genes in Alzheimer’s disease. Curr. Opin. Neurol..

[101] Daskoulidou N., Shaw B., Zelek W.M., Morgan B.P. (2025). The Alzheimer’s disease-associated complement receptor 1 variant confers risk by impacting glial phagocytosis. Alzheimer’s Dement..

[102] Mattsson-Carlgren N., Grinberg L.T., Boxer A., Ossenkoppele R., Jonsson M., Seeley W., Ehrenberg A., Spina S., Janelidze S., Rojas-Martinex J. (2022). Cerebrospinal fluid biomarkers in autopsy-confirmed Alzheimer disease and frontotemporal lobar degeneration. Neurology.

[103] Kac P.R., González-Ortiz F., Emeršič A., Dulewicz M., Koutarapu S., Turton M., An Y., Smirnov D., Kulczyńska-Przybik A., Varma V.R. (2024). Plasma p-tau212 antemortem diagnostic performance and prediction of autopsy verification of Alzheimer’s disease neuropathology. Nat. Commun..

[104] Liu Y., Kwok W., Yoon H., Ryu J.C., Stevens P., Hawkinson T.R., Shedlock C.J., Ribas R.A., Medina T., Keohane S.B. (2024). Imbalance in Glucose Metabolism Regulates the Transition of Microglia from Homeostasis to Disease-Associated Microglia Stage 1. J. Neurosci..

[105] Minter M.R., Taylor J.M., Crack P.J. (2016). The contribution of neuroinflammation to amyloid toxicity in Alzheimer’s disease. J. Neurochem..

[106] de Mello J.E., Teixeira F.C., dos Santos A., Luduvico K., Soares de Aguiar M.S., Domingues W.B., Campos V.F., Tavares R.G., Schneider A., Stefanello F.M. (2024). Treatment with Blackberry Extract and Metformin in Sporadic Alzheimer’s Disease Model: Impact on Memory, Inflammation, Redox Status, Phosphorylated Tau Protein and Insulin Signaling. Mol. Neurobiol..

[107] Thakur S., Dhapola R., Sarma P., Medhi B., Reddy D.H.K. (2023). Neuroinflammation in Alzheimer’s Disease: Current Progress in Molecular Signaling and Therapeutics. Inflammation.

[108] Van Gijsel-Bonnello M., Baranger K., Benech P., Rivera S., Khrestchatisky M., De Reggi M., Gharib B. (2017). Metabolic changes and inflammation in cultured astrocytes from the 5xFAD mouse model of Alzheimer’s disease: Alleviation by pantethine. PLoS ONE.

[109] van der Harg J.M., Eggels L., Ruigrok S.R., Jeroen J.J., La Fleur S.E., Scheper W. (2015). Neuroinflammation is not a prerequisite for diabetes-induced Tau phosphorylation. Front. Neurosci..

[110] Kacířová M., Železná B., Blažková M., Holubová M., Popelová A., Kuneš J., Šedivá B., Maletínská L. (2021). Aging and high-fat diet feeding lead to peripheral insulin resistance and sex-dependent changes in brain of mouse model of tau pathology THY-Tau22. J. Neuroinflamm..

[111] Dolatshahi M., Commean P.K., Rahmani F., Liu J., Lloyd L.K., Nguyen C., Hantler N., Ly M., Yu G., Ippolito J.E. (2024). Alzheimer Disease Pathology and Neurodegeneration in Midlife Obesity: A Pilot Study. Aging Dis..

[112] Marques F., Sousa J.C., Sousa N., Palha J.A. (2013). Blood-brain-barriers in aging and in Alzheimer’s disease. Mol. Neurodegener..

[113] Sagare A.P., Winkler Ea Bell R.D., Deane R., Zlokovic B.V. (2011). From the liver to the blood-brain barrier: An interconnected system regulating brain amyloid-β levels. J. Neurosci. Res..

[114] Erickson M.A., Banks W.A. (2013). Blood-brain barrier dysfunction as a cause and consequence of Alzheimer’s disease. J. Cereb. Blood Flow Metab..

[115] Dorrance A.M., Matin N., Pires P.W. (2014). The Effects of Obesity on the Cerebral Vasculature. Curr. Vasc. Pharmacol..

[116] Kim J.A., Montagnani M., Kwang K.K., Quon M.J. (2006). Reciprocal relationships between insulin resistance and endothelial dysfunction: Molecular and pathophysiological mechanisms. Circulation.

[117] Macvicar B.A., Newman E.A. (2015). Astrocyte Regulation of Blood Flow in the Brain. Cold Spring Harb. Perspect. Biol..

[118] Kisler K., Nelson A.R., Rege S.V., Ramanathan A., Wang Y., Ahuja A., Lazic D., Tsai P.S., Zhao Z., Zhou Y. (2017). Pericyte degeneration leads to neurovascular uncoupling and limits oxygen supply to brain. Nat. Neurosci..

[119] Hotamisligil G.S. (2017). Inflammation, metaflammation and immunometabolic disorders. Nature.

[120] de Paula G.C., Brunetta H.S., Engel D.F., Gaspar J.M., Velloso L.A., Engblom D., de Oliveira J., de Bem A.F. (2021). Hippocampal Function Is Impaired by a Short-Term High-Fat Diet in Mice: Increased Blood–Brain Barrier Permeability and Neuroinflammation as Triggering Events. Front. Neurosci..

[121] Wang X.L., Li L. (2021). Microglia Regulate Neuronal Circuits in Homeostatic and High-Fat Diet-Induced Inflammatory Conditions. Front. Cell. Neurosci..

[122] Beilharz J.E., Maniam J., Morris M.J. (2015). Diet-Induced Cognitive Deficits: The Role of Fat and Sugar, Potential Mechanisms and Nutritional Interventions. Nutrients.

[123] Molteni R., Barnard R.J., Ying Z., Roberts C.K., Gómez-Pinilla F. (2002). A High-Fat, Refined Sugar Diet Reduces Hippocampal Brain-Derived Neurotrophic Factor, Neuronal Plasticity, and Learning. Neuroscience.

[124] Barron A.M., Tokunaga M., Zhang M.R., Ji B., Suhara T., Higuchi M. (2016). Assessment of neuroinflammation in a mouse model of obesity and β-amyloidosis using PET. J. Neuroinflamm..

[125] Rudge J.D.A. (2022). A New Hypothesis for Alzheimer’s Disease: The Lipid Invasion Model. J. Alzheimer’s Dis. Rep..

[126] Gong Z., Pan J., Shen Q., Li M., Peng Y. (2018). Mitochondrial dysfunction induces NLRP3 inflammasome activation during cerebral ischemia/reperfusion injury. J. Neuroinflamm..

[127] Xu X., Pang Y., Fan X. (2025). Mitochondria in oxidative stress, inflammation and aging: From mechanisms to therapeutic advances. Signal Transduct. Target. Ther..

[128] Henn R.E., Elzinga S.E., Glass E., Parent R., Guo K., Allouch A.A., Mendelson F.E., Hayes J., Webber-Davis I., Murphy G.G. (2022). Obesity-induced neuroinflammation and cognitive impairment in young adult versus middle-aged mice. Immun. Ageing.

[129] González Olmo B.M., Bettes M.N., DeMarsh J.W., Zhao F., Askwith C., Barrientos R.M. (2023). Short-term high-fat diet consumption impairs synaptic plasticity in the aged hippocampus via IL-1 signaling. NPJ Sci. Food.

[130] Képes Z., Aranyi C., Forgács A., Nagy F., Kukuts K., Hascsi Z., Esze R., Somodi S., Káplár M., Varga J. (2021). Glucose-level dependent brain hypometabolism in type 2 diabetes mellitus and obesity. Eur. J. Hybrid Imaging.

[131] Pegueroles J., Pané A., Vilaplana E., Montal V., Bejanin A., Videla L., Carmona-Iragui M., Barroeta I., Ibarzabal A., Casajoana A. (2020). Obesity impacts brain metabolism and structure independently of amyloid and tau pathology in healthy elderly. Alzheimer’s Dement. Diagn. Assess. Dis. Monit..

[132] Wang H., Kulas J.A., Wang C., Holtzman D.M., Ferris H.A., Hansen S.B. (2021). Regulation of beta-amyloid production in neurons by astrocyte-derived cholesterol. Proc. Natl. Acad. Sci. USA.

[133] Montague-Cardoso K. (2021). Beta-amyloid production in neurons is regulated by astrocyte-derived cholesterol. Commun. Biol..

[134] Boccara E., Golan S., Beeri M.S. (2023). The association between regional adiposity, cognitive function, and dementia-related brain changes: A systematic review. Front. Med..

[135] Arvanitakis Z., Grodstein F., Bienias J.L., Schneider J.A., Wilson R.S., Kelly J.F., Evans D.A., Bennett D.A. (2008). Relation of NSAIDs to incident AD, change in cognitive function, and AD pathology. Neurology.

[136] Ostrowski K., Rohde T., Asp S., Schjerling P., Pedersen B.K. (1999). Pro- and anti-inflammatory cytokine balance in strenuous exercise in humans. J. Physiol..

[137] Vieira V.J., Valentine R.J., Wilund K.R., Antao N., Baynard T., Woods J.A. (2009). Effects of exercise and low-fat diet on adipose tissue inflammation and metabolic complications in obese mice. Am. J. Physiol. Endocrinol. Metab..

[138] Ng R.C.L., Jian M., Ma O.K.F., Bunting M., Kwan J.S.C., Zhou G.J., Senthilkumar K., Iyaswamy A., Chan P.K., Li M. (2021). Chronic oral administration of adipoRon reverses cognitive impairments and ameliorates neuropathology in an Alzheimer’s disease mouse model. Mol. Psychiatry.

[139] Cui W., Sun C., Ma Y., Wang S., Wang X., Zhang Y. (2020). Inhibition of TLR4 Induces M2 Microglial Polarization and Provides Neuroprotection via the NLRP3 Inflammasome in Alzheimer’s Disease. Front. Neurosci..

[140] Arnoldussen I.A.C., Kiliaan A.J., Gustafson D.R. (2014). Obesity and dementia: Adipokines interact with the brain. Eur. Neuropsychopharmacol..

[141] Calió M.L., Mosini A.C., Marinho D.S., Salles G.N., Massinhani F.H., Ko G.M., Porcionatto M.A. (2021). Leptin enhances adult neurogenesis and reduces pathological features in a transgenic mouse model of Alzheimer’s disease. Neurobiol. Dis..

[142] Song J., Kang S.M., Kim E., Kim C.H., Song H.T., Lee J.E. (2015). Adiponectin receptor-mediated signaling ameliorates cerebral cell damage and regulates the neurogenesis of neural stem cells at high glucose concentrations: An in vivo and in vitro study. Cell Death Dis..

[143] Abdelwahed O.M., Tork O.M., Gamal el Din M.M., Rashed L., Zickri M. (2018). Effect of glucagon-like peptide-1 analogue; Exendin-4, on cognitive functions in type 2 diabetes mellitus; possible modulation of brain derived neurotrophic factor and brain Visfatin. Brain Res. Bull..

[144] Cisternas P., Gherardelli C., Gutierrez J., Salazar P., Mendez-Orellana C., Wong G.W., Inestrosa N.C. (2023). Adiponectin and resistin modulate the progression of Alzheimer’s disease in a metabolic syndrome model. Front. Endocrinol..

[145] Lloyd J.W., Zerfass K.M., Heckstall E.M., Evans K.A. (2015). Diet-induced increases in chemerin are attenuated by exercise and mediate the effect of diet on insulin and HOMA-IR. Ther. Adv. Endocrinol. Metab..

[146] Chen Y., Liu Z., Gong P., Zhang H., Chen Y., Yao S., Li W., Zhang Y., Yu Y. (2022). The Chemerin/CMKLR1 Axis Is Involved in the Recruitment of Microglia to Aβ Deposition through p38 MAPK Pathway. Int. J. Mol. Sci..

[147] Bao H., Yang X., Huang Y.X., Qiu H., Huang G., Xiao H., Kuai J. (2016). The neuroprotective effect of apelin-13 in a mouse model of intracerebral hemorrhage. Neurosci. Lett..

[148] Zhou H., Zhang Z., Qian G., Zhou J. (2020). Omentin-1 attenuates adipose tissue inflammation via restoration of TXNIP/NLRP3 signaling in high-fat diet-induced obese mice. Fundam. Clin. Pharmacol..

[149] Yang E., Cai Y., Yao X., Liu J., Wang Q., Jin W., Wu Q., Fan W., Qiu L., Kang C. (2019). Tissue plasminogen activator disrupts the blood-brain barrier through increasing the inflammatory response mediated by pericytes after cerebral ischemia. Aging.

[150] Wołoszynowska-Fraser M.U., Rossi S.L., Long J.M., McCaffery P.J., Rapp P.R. (2021). Differential retinoic acid signaling in the hippocampus of aged rats with and without memory impairment. eNeuro.

[151] Jung C.H., Lee M.J., Kang Y.M., Lee Y.L., Yoon H.K., Kang S.W., Lee W.J., Park J.Y. (2014). Vaspin inhibits cytokine-induced nuclear factor-kappa B activation and adhesion molecule expression via AMP-activated protein kinase activation in vascular endothelial cells. Cardiovasc. Diabetol..

[152] Gaweda-Walerych K., Aragona V., Lodato S., Sitek E.J., Narożańska E., Buratti E. (2025). Progranulin deficiency in the brain: The interplay between neuronal and non-neuronal cells. Transl. Neurodegener..

[153] Myers M.G., Cowley M.A., Münzberg H. (2008). Mechanisms of Leptin Action and Leptin Resistance. Annu. Rev. Physiol..

[154] Flak J.N., Myers M.G. (2016). Minireview: CNS Mechanisms of Leptin Action. Mol. Endocrinol..

[155] Nazarians-Armavil A., Menchella J.A., Belsham D.D. (2013). Cellular Insulin Resistance Disrupts Leptin-Mediated Control of Neuronal Signaling and Transcription. Mol. Endocrinol..

[156] Tezapsidis N., Johnston J.M., Smith M.A., Ashford J.W., Casadesus G., Robakis N.K., Wolozin B., Perry G., Zhu X., Greco S.J. (2009). Leptin: A novel therapeutic strategy for Alzheimer’s disease. J. Alzheimer’s Dis..

[157] Bonda D.J., Stone J.G., Torres S.L., Siedlak S.L., Perry G., Kryscio R., Jicha G., Casadesus G., Smith M.A., Zhu X. (2014). Dysregulation of leptin signaling in Alzheimer disease: Evidence for neuronal leptin resistance. J. Neurochem..

[158] Magalhães C.A., Carvalho M.G., Sousa L.P., Caramelli P., Gomes K.B. (2015). Leptin in Alzheimer’s disease. Clin. Chim. Acta.

[159] Bulló M., García-Lorda P., Megias I., Salas-Salvadó J. (2003). Systemic inflammation, adipose tissue tumor necrosis factor, and leptin expression. Obes. Res..

[160] Ahima R.S. (2010). Connecting Leptin and Alzheimer Disease. Arch. Neurol..

[161] Lee S., Byun M.S., Yi D., Ahn H., Jung G., Jung J.H., Chang Y.Y., Kim K., Choi H., Choi J. (2024). Plasma Leptin and Alzheimer Protein Pathologies Among Older Adults. JAMA Netw. Open.

[162] Mee-Inta O., Zhao Z.W., Kuo Y.M. (2019). Physical Exercise Inhibits Inflammation and Microglial Activation. Cells.

[163] Chan K.H., Lam K.S.L., Cheng O.Y., Kwan J.S.C., Ho P.W.L., Cheng K.K.Y., Chung S.K., Ho J.W.M., Guo V.Y., Xu A. (2012). Adiponectin is Protective against Oxidative Stress Induced Cytotoxicity in Amyloid-Beta Neurotoxicity. PLoS ONE.

[164] Jian M., Kwan J.S.C., Bunting M., Ng R.C.L., Chan K.H. (2019). Adiponectin suppresses amyloid-β oligomer (AβO)-induced inflammatory response of microglia via AdipoR1-AMPK-NF-κB signaling pathway. J. Neuroinflamm..

[165] Ng R.C.L., Chan K.H. (2017). Potential neuroprotective effects of adiponectin in Alzheimer’s disease. Int. J. Mol. Sci..

[166] Shi Y., Zhu N., Qiu Y., Tan J., Wang F., Qin L., Dai A. (2023). Resistin-like molecules: A marker, mediator and therapeutic target for multiple diseases. Cell Commun. Signal..

[167] Bokarewa M., Nagaev I., Dahlberg L., Smith U., Tarkowski A. (2005). Resistin, an Adipokine with Potent Proinflammatory Properties. J. Immunol..

[168] Schwartz D.R., Lazar M.A. (2011). Human resistin: Found in translation from mouse to man. Trends Endocrinol. Metab..

[169] Taouis M., Benomar Y. (2021). Is resistin the master link between inflammation and inflammation-related chronic diseases?. Mol. Cell. Endocrinol..

[170] Amine H., Benomar Y., Taouis M. (2021). Palmitic acid promotes resistin-induced insulin resistance and inflammation in SH-SY5Y human neuroblastoma. Sci. Rep..

[171] Tripathi D., Kant S., Pandey S., Ehtesham N.Z. (2020). Resistin in metabolism, inflammation, and disease. FEBS J..

[172] Li S., Han X., Song J., Dong M., Xie T. (2024). Mechanism of Action and Risk Prediction of Adiponectin in Cardiovascular Diseases. Front. Biosci.—Landmark.

[173] Jamaluddin M.S., Yan S., Lü J., Liang Z., Yao Q., Chen C. (2013). Resistin Increases Monolayer Permeability of Human Coronary Artery Endothelial Cells. PLoS ONE.

[174] Salameh T.S., Mortell W., Banks W.A. (2018). Resistin Is Associated with Blood-Brain Barrier Disruption in Mice Resistant to Diet-Induced Obesity and Treated with Topiramate. Diabetes.

[175] Mounien L., Marty N., Tarussio D., Metref S., Genoux D., Preitner F., Foretz M., Thorens B. (2010). Glut2-dependent glucose-sensing controls thermoregulation by enhancing the leptin sensitivity of NPY and POMC neurons. FASEB J.

[176] Kahn B.B., Minokoshi Y. (2013). Leptin, GABA, and glucose control. Cell Metab..

[177] Varela L., Horvath T.L. (2012). Leptin and insulin pathways in POMC and AgRP neurons that modulate energy balance and glucose homeostasis. EMBO Rep..

[178] Zhang D., Wang X., Lu X.Y. (2016). Adiponectin exerts neurotrophic effects on dendritic arborization, spinogenesis, and neurogenesis of the dentate gyrus of male mice. Endocrinology.

[179] Yamauchi T., Kamon J., Minokoshi Y., Ito Y., Waki H., Uchida S., Yamashita S., Noda M., Kita S., Ueki K. (2002). Adiponectin stimulates glucose utilization and fatty-acid oxidation by activating AMP-activated protein kinase. Nat. Med..

[180] Yan X.D., Qu X.S., Yin J., Qiao J., Zhang J., Qi J.S., Wu M.N. (2022). Adiponectin Ameliorates Cognitive Behaviors and in vivo Synaptic Plasticity Impairments in 3xTg-AD Mice. J. Alzheimer’s Dis..

[181] Hallschmid M. (2021). Intranasal Insulin for Alzheimer’s Disease. CNS Drugs.

[182] Kastin A.J., Pan W. (2006). Intranasal Leptin: Blood-Brain Barrier Bypass (BBBB) for Obesity?. Endocrinology.

[183] Prévost M., Crépin D., Rifai SAl Poizat G., Gonçalves M., van Barneveld F., Shadpay R., Taouis K., Riffault L., Benomar Y., Taouis M. (2025). The Resistin/TLR4/miR-155-5p axis: A novel signaling pathway in the onset of hypothalamic neuroinflammation. J. Neuroinflamm..

[184] Zhu Y., Wan N., Shan X., Deng G., Xu Q., Ye H., Sun Y. (2021). Celastrol targets adenylyl cyclase-associated protein 1 to reduce macrophages-mediated inflammation and ameliorates high fat diet-induced metabolic syndrome in mice. Acta Pharm. Sin. B.

[185] Li R., Lau W.B., Ma X.L. (2010). Adiponectin resistance and vascular dysfunction in the hyperlipidemic state. Acta Pharmacol. Sin..

[186] Kaiyrlykyzy A., Umbayev B., Masoud A.R., Baibulatova A., Tsoy A., Olzhayev F., Alzhanova D., Zholdasbekova G., Davletov K., Akilzhanova A. (2022). Circulating adiponectin levels, expression of adiponectin receptors, and methylation of adiponectin gene promoter in relation to Alzheimer’s disease. BMC Med. Genom..

[187] Hui E.K., Mukadam N., Kohl G., Livingston G. (2025). Effect of diabetes medications on the risk of developing dementia, mild cognitive impairment, or cognitive decline: A systematic review and meta-analysis. J. Alzheimer’s Dis..

[188] Prokopidis K., Daly R.M., Suetta C. (2025). Weighing the risk of GLP-1 treatment in older adults: Should we be concerned about sarcopenic obesity?. J. Nutr. Health Aging.

[189] Carvalho A.F., Rocha D.Q.C., McIntyre R.S., Mesquita L.M., Köhler C.A., Hyphantis T.N., Sales P.M.G., Machado-Vieira R., Berk M. (2014). Adipokines as emerging depression biomarkers: A systematic review and meta-analysis. J. Psychiatr. Res..

[190] Letra L., Matafome P., Rodrigues T., Duro D., Lemos R., Baldeiras I., Patrcio M., Castelo-Branco M., Caetano G., Seica R. (2019). Association between adipokines and biomarkers of Alzheimer’s disease: A cross-sectional study. J. Alzheimer’s Dis..

[191] Kim K.Y., Ha J., Kim M., Cho S.Y., Kim H., Kim E. (2022). Plasma adiponectin levels predict cognitive decline and cortical thinning in mild cognitive impairment with beta-amyloid pathology. Alzheimers Res. Ther..

[192] Gorska-Ciebiada M., Saryusz-Wolska M., Borkowska A., Ciebiada M., Loba J. (2015). Adiponectin, leptin and IL-1 β in elderly diabetic patients with mild cognitive impairment. Metab. Brain Dis..

[193] Zeki Al Hazzouri A., Stone K.L., Haan M.N., Yaffe K. (2013). Leptin, mild cognitive impairment, and dementia among elderly women. J. Gerontol.—Ser. A Biol. Sci. Med. Sci..

[194] Olszanecka-Glinianowicz M., Owczarek A., Bozentowicz-Wikarek M., Brzozowska A., Mossakowska M., Zdrojewski T., Grodzicki T., Więcek A., Chudek J. (2014). Relationship between circulating visfatin/NAMPT, nutritional status and insulin resistance in an elderly population—Results from the PolSenior substudy. Metabolism.

[195] Guo D., Yuan Y., Huang R., Tian S., Wang J., Lin H., An K., Han J., Wang S. (2019). Association between plasma adipsin level and mild cognitive impairment in Chinese patients with type 2 diabetes: A cross-sectional study. BMC Endocr. Disord..

[196] Vaňková M., Vacínová G., Včelák J., Vejražková D., Lukášová P., Rusina R., Holmerová I., Jarolímová E., Vaňková H., Bendlová B. (2020). Plasma Levels of Adipokines in Patients with Alzheimer’s Disease—Where Is the “Breaking Point” in Alzheimer’s Disease Pathogenesis?. Physiol. Res..

[197] Cisternas P., Martinez M., Ahima R.S., William Wong G., Inestrosa N.C. (2019). Modulation of Glucose Metabolism in Hippocampal Neurons by Adiponectin and Resistin. Mol. Neurobiol..

[198] Fernández C.M., Moltó E., Gallardo N., del Arco A., Martínez C., Andrés A., Ros M., Carrascosa J.M., Arribas C. (2009). The expression of rat resistin isoforms is differentially regulated in visceral adipose tissues: Effects of aging and food restriction. Metabolism.

[199] Kos K., Harte A.L., da Silva N.F., Tonchev A., Chaldakov G., James S., Snead D.R., Hoggart B., O’Hare J.P., McTernan P.G. (2007). Adiponectin and resistin in human cerebrospinal fluid and expression of adiponectin receptors in the human hypothalamus. J. Clin. Endocrinol. Metab..

[200] Benomar Y., Gertler A., De Lacy P., Crépin D., Hamouda H.O., Riffault L., Taouis M. (2013). Central resistin overexposure induces insulin resistance through toll-like receptor 4. Diabetes.

[201] Lauretti E., Dincer O., Praticò D. (2020). Glycogen synthase kinase-3 signaling in Alzheimer’s disease. Biochim. Biophys. Acta (BBA)—Mol. Cell Res..

[202] Lehmann S., Schraen-Maschke S., Buée L., Vidal J.S., Delaby C., Hirtz C., Blanc F., Paquet C., Allinquant B., Bombois S. (2024). Clarifying the association of CSF Aβ, tau, BACE1, and neurogranin with AT(N) stages in Alzheimer disease. Mol. Neurodegener..

[203] Rosenberg G.A. (2016). Matrix Metalloproteinase-Mediated Neuroinflammation in Vascular Cognitive Impairment of the Binswanger Type. Cell. Mol. Neurobiol..

[204] Greene C., Hanley N., Campbell M. (2019). Claudin-5: Gatekeeper of neurological function. Fluids Barriers CNS.

[205] Thundyil J., Pavlovski D., Sobey C.G., Arumugam T.V. (2012). Adiponectin receptor signalling in the brain. Br. J. Pharmacol..

[206] Samant N.P., Gupta G.L. (2021). Adiponectin: A potential target for obesity-associated Alzheimer’s disease. Metab. Brain Dis..

[207] Vu V., Bui P., Eguchi M., Xu A., Sweeney G. (2013). Globular adiponectin induces LKB1/AMPK-dependent glucose uptake via actin cytoskeleton remodeling. J. Mol. Endocrinol..

[208] Takahashi J., Takahashi N., Tadaishi M., Shimizu M., Kobayashi-Hattori K. (2022). Valerenic Acid Promotes Adipocyte Differentiation, Adiponectin Production, and Glucose Uptake via Its PPARγLigand Activity. ACS Omega.

[209] Novinbahador T., Abbasi A., Molani-Gol R., Aghebati-Maleki L., Pouraghaei A., Soleimanpour H. (2025). Neuroprotection through adiponectin receptor agonist: An updated meta-analysis of preclinical Alzheimer’s disease studies. BMC Neurol..

[210] Ng R.C.L., Cheng O.Y., Jian M., Kwan J.S.C., Ho P.W.L., Cheng K.K.Y., Yeung P.K.K., Zhou L.L., Hoo R.L.C., Chung S.K. (2016). Chronic adiponectin deficiency leads to Alzheimer’s disease-like cognitive impairments and pathologies through AMPK inactivation and cerebral insulin resistance in aged mice. Mol. Neurodegener..

[211] He K., Nie L., Ali T., Wang S., Chen X., Liu Z., Li W., Zhang K., Xu J., Liu J. (2021). Adiponectin alleviated Alzheimer-like pathologies via autophagy-lysosomal activation. Aging Cell.

[212] Chen R.J., Shu Y., Zeng Y. (2020). Links Between Adiponectin and Dementia: From Risk Factors to Pathophysiology. Front. Aging Neurosci..

[213] Sindzingre L., Bouaziz-Amar E., Mouton-Liger F., Cognat E., Dumurgier J., Vrillon A., Paquet C., Lilamand M. (2024). The role of adiponectin in Alzheimer’s disease: A translational review. J. Nutr. Health Aging.

[214] Degawa-Yamauchi M., Bovenkerk J.E., Juliar B.E., Watson W., Kerr K., Jones R., Zhu Q., Considine R.V. (2003). Serum Resistin (FIZZ3) Protein Is Increased in Obese Humans. J. Clin. Endocrinol. Metab..

[215] Inadera H. (2008). The usefulness of circulating adipokine levels for the assessment of obesity-related health problems. Int. J. Med. Sci..

[216] Clain J., Couret D., Planesse C., Krejbich-Trotot P., Meilhac O., Lefebvre D’Hellencourt C., Viranaicken W., Diotel N. (2022). Distribution of Adiponectin Receptors in the Brain of Adult Mouse: Effect of a Single Dose of the Adiponectin Receptor Agonist, AdipoRON, on Ischemic Stroke. Brain Sci..

[217] Lee S., Lee H.C., Kwon Y.W., Lee S.E., Cho Y., Kim J., Lee S., Kim J.Y., Lee J., Yang H.M. (2014). Adenylyl cyclase-associated protein 1 is a receptor for human resistin and mediates inflammatory actions of human monocytes. Cell Metab..

[218] Nicolas S., Cazareth J., Zarif H., Guyon A., Heurteaux C., Chabry J., Petit-Paitel A. (2017). Globular adiponectin limits microglia pro-inflammatory phenotype through an AdipoR1/NF-κB signaling pathway. Front. Cell. Neurosci..

[219] Al Hannan F., Culligan K.G. (2015). Human resistin and the RELM of Inflammation in diabesity. Diabetol. Metab. Syndr..

[220] Kadowaki T., Yamauchi T., Kubota N., Hara K., Ueki K., Tobe K. (2006). Adiponectin and adiponectin receptors in insulin resistance, diabetes, and the metabolic syndrome. J. Clin. Investig..

[221] Song J., Choi S.M., Whitcomb D.J., Kim B.C. (2017). Adiponectin controls the apoptosis and the expression of tight junction proteins in brain endothelial cells through AdipoR1 under beta amyloid toxicity. Cell Death Dis..

[222] Ali T., Rehman S.U., Khan A., Badshah H., Abid NBin Kim M.W., Jo M.H., Chung S.S., Lee Hgon Rutten B.P.F., Kim M.O. (2021). Adiponectin-mimetic novel nonapeptide rescues aberrant neuronal metabolic-associated memory deficits in Alzheimer’s disease. Mol. Neurodegener..

[223] Balasubramanian P., Schaar A.E., Gustafson G.E., Smith A.B., Howell P.R., Greenman A., Baum S., Colman R.J., Lamming D.W., Diffee G.M. (2022). Adiponectin receptor agonist AdipoRon improves skeletal muscle function in aged mice. Elife.

[224] Wennberg A.M.V., Gustafson D., Hagen C.E., Roberts R.O., Knopman D., Jack C., Petersen R.C., Mielke M.M. (2016). Serum Adiponectin Levels, Neuroimaging, and Cognition in the Mayo Clinic Study of Aging. J. Alzheimer’s Dis..

[225] Habib S.S., Al-Khlaiwi T., Butt M.A., Habib S.M., Al-Khliwi H., Al-Regaiey K. (2023). Novel Adiponectin-Resistin Indices and Ratios Predict Increased Cardiovascular Risk in Patients with Type 2 Diabetes Mellitus. J. Saudi Heart Assoc..

[226] Zhou J., Kim Y.K., Li C., Park S. (2025). Natural compounds for Alzheimer’s prevention and treatment: Integrating SELFormer-based computational screening with experimental validation. Comput. Biol. Med..

[227] Aktary N., Jeong Y., Oh S., Shin Y., Sung Y., Rahman M., Ramos Santiago L., Choi J., Song H.G., Nurkolis F. (2025). Unveiling the therapeutic potential of natural products in Alzheimer’s disease: Insights from in vitro, in vivo, and clinical studies. Front. Pharmacol..

[228] Yang J., Shi X., Wang Y., Ma M., Liu H., Wang J., Xu Z. (2023). Multi-Target Neuroprotection of Thiazolidinediones on Alzheimer’s Disease via Neuroinflammation and Ferroptosis. J. Alzheimer’s Dis..

[229] Zhao Z., Yan J., Huang L., Yang X. (2024). Phytochemicals targeting Alzheimer’s disease via the AMP-activated protein kinase pathway, effects, and mechanisms of action. Biomed. Pharmacother..

[230] Deepika Maurya P.K. (2022). Health Benefits of Quercetin in Age-Related Diseases. Molecules.

[231] Cheng M., Yuan C., Ju Y., Liu Y., Shi B., Yang Y., Jin S., He X., Zhang L., Min D. (2024). Quercetin Attenuates Oxidative Stress and Apoptosis in Brain Tissue of APP/PS1 Double Transgenic AD Mice by Regulating Keap1/Nrf2/HO-1 Pathway to Improve Cognitive Impairment. Behav. Neurol..

[232] Rezvan N., Moini A., Gorgani-Firuzjaee S., Hosseinzadeh-Attar M.J. (2018). Oral quercetin supplementation enhances adiponectin receptor transcript expression in polycystic ovary syndrome patients: A randomized placebo-controlled double-blind clinical trial. Cell J..

[233] Zhong X., Liu M., Yao W., Du K., He M., Jin X., Jiao L., Ma G., Wei B., Wei M. (2019). Epigallocatechin-3-Gallate Attenuates Microglial Inflammation and Neurotoxicity by Suppressing the Activation of Canonical and Noncanonical Inflammasome via TLR4/NF-κB Pathway. Mol. Nutr. Food Res..

[234] Shimada M., Mochizuki K., Sakurai N., Goda T. (2007). Dietary supplementation with epigallocatechin gallate elevates levels of circulating adiponectin in non-obese type-2 diabetic Goto-Kakizaki rats. Biosci. Biotechnol. Biochem..

[235] Park D.J., Kang JBin Koh P.O. (2024). Epigallocatechin gallate improves neuronal damage in animal model of ischemic stroke and glutamate-exposed neurons via modulation of hippocalcin expression. PLoS ONE.

[236] Quadros Gomes B.A., Bastos Silva J.P., Rodrigues Romeiro C.F., dos Santos S.M., Rodrigues C.A., Gonçalves P.R., Sakai J.T., Santos Mendes P.F., Pompeu Varela E.L., Monteiro M.C. (2018). Neuroprotective Mechanisms of Resveratrol in Alzheimer’s Disease: Role of SIRT1. Oxidative Med. Cell. Longev..

[237] Veronica Witte A., Kerti L., Margulies D.S., Flöel A. (2014). Effects of Resveratrol on Memory Performance, Hippocampal Functional Connectivity, and Glucose Metabolism in Healthy Older Adults. J. Neurosci..

[238] Jeon B.T., Jeong E.A., Shin H.J., Lee Y., Lee D.H., Kim H.J., Kang S.S., Cho G.J., Choi W.S., Roh G.S. (2012). Resveratrol attenuates obesity-associated peripheral and central inflammation and improves memory deficit in mice fed a high-fat diet. Diabetes.

[239] Ege D. (2021). Action Mechanisms of Curcumin in Alzheimer’s Disease and Its Brain Targeted Delivery. Materials.

[240] Teter B., Morihara T., Lim G.P., Chu T., Jones M.R., Zuo X., Paul R.M., Frautschy S.A., Cole G.M. (2019). Curcumin restores innate immune Alzheimer’s disease risk gene expression to ameliorate Alzheimer pathogenesis. Neurobiol. Dis..

[241] Panahi Y., Khalili N., Sahebi E., Namazi S., Atkin S.L., Majeed M., Sahebkar A. (2018). Curcuminoids Plus Piperine Modulate Adipokines in Type 2 Diabetes Mellitus. Curr. Clin. Pharmacol..

[242] Zhang H., Su Y., Sun Z., Chen M., Han Y., Li Y., Dong X., Ding S., Fang Z., Li W. (2021). Ginsenoside Rg1 alleviates Aβ deposition by inhibiting NADPH oxidase 2 activation in APP/PS1 mice. J. Ginseng Res..

[243] Yu X., Ye L., Zhang H., Zhao J., Wang G., Guo C., Shang W. (2014). Ginsenoside Rb1 ameliorates liver fat accumulation by upregulating perilipin expression in adipose tissue of db/db obese mice. J. Ginseng Res..

[244] Li Y., Zhang S., Zhu Z., Zhou R., Xu P., Zhou L., Kan Y., Li J., Zhao J., Fang P. (2022). Upregulation of adiponectin by Ginsenoside Rb1 contributes to amelioration of hepatic steatosis induced by high fat diet. J. Ginseng Res..

[245] Babalola J.A., Lang M., George M., Stracke A., Tam-Amersdorfer C., Itxaso I., Lucija D., Tadic J., Schilcher I., Loeffler T. (2023). Astaxanthin enhances autophagy, amyloid beta clearance and exerts anti-inflammatory effects in in vitro models of Alzheimer’s disease-related blood brain barrier dysfunction and inflammation. Brain Res..

[246] Han J.H., Lee Y.S., Im J.H., Ham Y.W., Lee H.P., Han S.B., Hong J.T. (2019). Astaxanthin ameliorates lipopolysaccharide-induced neuroinflammation, oxidative stress and memory dysfunction through inactivation of the signal transducer and activator of transcription 3 pathway. Mar. Drugs.

[247] Nawaz A., Nishida Y., Takikawa A., Fujisaka S., Kado T., Aminuddin A., Bilal M., Jeelani I., Aslam M.R., Nishimura A. (2021). Astaxanthin, a marine carotenoid, maintains the tolerance and integrity of adipose tissue and contributes to its healthy functions. Nutrients.

[248] Xiao M., Xiang W., Chen Y., Peng N., Du X., Lu S., Zuo Y., Li B., Hu Y., Li X. (2022). DHA Ameliorates Cognitive Ability, Reduces Amyloid Deposition, and Nerve Fiber Production in Alzheimer’s Disease. Front. Nutr..

[249] de Barbosa M.M.A.L., de Melo A.L.T.R., Damasceno N.R.T. (2017). The benefits of ω-3 supplementation depend on adiponectin basal level and adiponectin increase after the supplementation: A randomized clinical trial. Nutrition.

[250] Wu L., Xia M., Duan Y., Zhang L., Jiang H., Hu X., Yan H., Zhang Y., Gu Y., Shi H. (2019). Berberine promotes the recruitment and activation of brown adipose tissue in mice and humans. Cell Death Dis..

[251] Hu Y., Zhang P., Wang X. (2025). Berberine Exerts Neuroprotective Effects in Alzheimer’s Disease by Switching Microglia M1/M2 Polarization Through PI3K-AKT Signaling. Physiol. Res..

[252] Lu J., Zhou W., Dou F., Wang C., Yu Z. (2021). TRPV1 sustains microglial metabolic reprogramming in Alzheimer’s disease. EMBO Rep..

[253] Wang J., Sun B.L., Xiang Y., Tian D.Y., Zhu C., Li W.W., Liu Y.H., Bu XLe Shen L.L., Jin W.S., Wang Z. (2020). Capsaicin consumption reduces brain amyloid-beta generation and attenuates Alzheimer’s disease-type pathology and cognitive deficits in APP/PS1 mice. Transl. Psychiatry.

[254] Jalili C., Kiani A., Gholami M., Bahrehmand F., Fakhri S., Kakehbaraei S., Kakebaraei S. (2023). Brain targeting based nanocarriers loaded with resveratrol in Alzheimer’s disease: A review. IET Nanobiotechnol..

[255] Pei J.J., Palanisamy C.P., Natarajan P.M., Umapathy V.R., Roy J.R., Srinivasan G.P., Panagal M., Jayaraman S. (2024). Curcumin-loaded polymeric nanomaterials as a novel therapeutic strategy for Alzheimer’s disease: A comprehensive review. Ageing Res. Rev..

[256] Bray J.J.H., Foster-Davies H., Salem A., Hoole A.L., Obaid D.R., Halcox J.P.J., Stephens J.W. (2021). Glucagon-like peptide-1 receptor agonists improve biomarkers of inflammation and oxidative stress: A systematic review and meta-analysis of randomised controlled trials. Diabetes Obes. Metab..

[257] Monney M., Jornayvaz F.R., Gariani K. (2023). GLP-1 receptor agonists effect on cognitive function in patients with and without type 2 diabetes. Diabetes Metab..

[258] Foretz M., Guigas B., Bertrand L., Pollak M., Viollet B. (2014). Metformin: From mechanisms of action to therapies. Cell Metab..

[259] Riera-Guardia N., Rothenbacher D. (2008). The effect of thiazolidinediones on adiponectin serum level: A meta-analysis. Diabetes Obes. Metab..

[260] Saunders A.M., Burns D.K., Gottschalk W.K. (2021). Reassessment of Pioglitazone for Alzheimer’s Disease. Front. Neurosci..

[261] Craft S., Baker L.D., Montine T.J., Minoshima S., Watson G.S., Claxton A., Arbuckle M., Callaghan M., Tsai E., Plymate S.R. (2012). Intranasal insulin therapy for Alzheimer disease and amnestic mild cognitive impairment: A pilot clinical trial. Arch. Neurol..

[262] Claxton A., Baker L.D., Wilkinson C.W., Trittschuh E.H., Chapman D., Watson G.S., Cholerton B., Plymate S.R., Arbuckle M., Craft S. (2013). Sex and ApoE genotype differences in treatment response to two doses of intranasal insulin in adults with mild cognitive impairment or Alzheimer’s disease. J. Alzheimer’s Dis..

[263] Rege S.D., Geetha T., Griffin G.D., Broderick T.L., Babu J.R. (2014). Neuroprotective effects of resveratrol in Alzheimer disease pathology. Front. Aging Neurosci..

[264] Tomé-Carneiro J., Gonzálvez M., Larrosa M., Yáñez-Gascón M.J., García-Almagro F.J., Ruiz-Ros J.A., Tomás-Barberán F.A., García-Conesa M.T., Espín J.C. (2013). Grape resveratrol increases serum adiponectin and downregulates inflammatory genes in peripheral blood mononuclear cells: A triple-blind, placebo-controlled, one-year clinical trial in patients with stable coronary artery disease. Cardiovasc. Drugs Ther..

[265] Turner R.S., Thomas R.G., Craft S., Van Dyck C.H., Mintzer J., Reynolds B.A., Brewer J.B., Rissman R.A., Raman R., Aisen P.S. (2015). A randomized, double-blind, placebo-controlled trial of resveratrol for Alzheimer disease. Neurology.

[266] Meng X., Gong Y., Xiao F., Cao Z., Zhuang Z., Yi X., Wang J., Feng R., Gong C., Ni P. (2025). Curcumin’s multi-target mechanisms in the treatment of Alzheimer’s disease and creative modification techniques. J. Alzheimer’s Dis..

[267] Kaur K., Kulkarni Y.A., Wairkar S. (2024). Exploring the potential of quercetin in Alzheimer’s Disease: Pharmacodynamics, Pharmacokinetics, and Nanodelivery systems. Brain Res..

[268] Cai Z., Wang C., Yang W. (2016). Role of berberine in Alzheimer’s disease. Neuropsychiatr. Dis. Treat..

[269] Li Y., Wang P., Zhuang Y., Lin H., Li Y., Liu L., Meng Q., Cui T., Liu J., Li Z. (2011). Activation of AMPK by berberine promotes adiponectin multimerization in 3T3-L1 adipocytes. FEBS Lett..

[270] Yoshida H., Yanai H., Ito K., Tomono Y., Koikeda T., Tsukahara H., Tada N. (2010). Administration of natural astaxanthin increases serum HDL-cholesterol and adiponectin in subjects with mild hyperlipidemia. Atherosclerosis.

[271] Liu N., Lyu X., Zhang X., Zhang F., Chen Y., Li G. (2023). Astaxanthin attenuates cognitive deficits in Alzheimer’s disease models by reducing oxidative stress via the SIRT1/PGC-1α signaling pathway. Cell Biosci..

[272] Gray B., Steyn F., Davies P.S.W., Vitetta L. (2013). Omega-3 fatty acids: A review of the effects on adiponectin and leptin and potential implications for obesity management. Eur. J. Clin. Nutr..

[273] Kang J.H., Tsuyoshi G., Le Ngoc H., Kim H.M., Tu T.H., Noh H.J., Kim C.S., Choe S.Y., Kawada T., Yoo H. (2011). Dietary capsaicin attenuates metabolic dysregulation in genetically obese diabetic mice. J. Med. Food.

[274] Forny-Germano L., De Felice F.G., Do Nascimento Vieira M.N. (2019). The role of leptin and adiponectin in obesity-associated cognitive decline and Alzheimer’s disease. Front. Neurosci..

[275] Cente M., Zorad S., Smolek T., Fialova L., Paulenka Ivanovova N., Krskova K., Balazova L., Skrabana R., Filipcik P. (2020). Plasma Leptin Reflects Progression of Neurofibrillary Pathology in Animal Model of Tauopathy. Cell. Mol. Neurobiol..

[276] McGuire M.J., Ishii M. (2016). Leptin Dysfunction and Alzheimer’s Disease: Evidence from Cellular, Animal, and Human Studies. Cell. Mol. Neurobiol..

[277] Ueda H., Howson J.M.M., Esposito L., Heward J., Snook H., Chamberlain G., Rainbow D.B., Hunter K.M.D., Smith A.N., Di Genova G. (2003). Association of the T-cell regulatory gene CTLA4 with susceptibility to autoimmune disease. Nature.

[278] Gil-Bea F.J., Solas M., Solomon A., Mugueta C., Winblad B., Kivipelto M., Ramirez M.J., Cedazo-Mínguez A. (2010). Insulin levels are decreased in the cerebrospinal fluid of women with prodomal alzheimer’s disease. J. Alzheimer’s Dis..

[279] Kempers M.J.E., Van Der Sluijs Veer L., Nijhuis-Van Der Sanden R.W.G., Lanting C.I., Kooistra L., Wiedijk B.M., Last B.F., De Vijlder J.J.M., Grootenhuis M.A., Vulsma T. (2007). Neonatal screening for congenital hypothyroidism in The Netherlands: Cognitive and motor outcome at 10 years of age. J. Clin. Endocrinol. Metab..

[280] Ishii M., Iadecola C. (2016). Adipocyte-derived factors in age-related dementia and their contribution to vascular and Alzheimer pathology. Biochim. Biophys. Acta Mol. Basis Dis..

